# Programme of the 21st European Conference on Eye Movements

**DOI:** 10.16910/jemr.15.5.1

**Published:** 2022-08-21

**Authors:** Victoria McGowan, Ascensión Pagán, Kevin B. Paterson, David Souto, Rudolf Groner

**Keywords:** eye movements, eye tracking, reading attention, Art perception, fixation, saccade

## Abstract

About ECEM

ECEM was initiated by Rudolf Groner (Bern), Dieter Heller (Bayreuth at the time) and Henk Breimer (Tilburg) in the 198 to provide a forum for an interdisciplinary group of scientists interested in eye movements. Since the inaugural meeting in Bern, the conference has been held every two years in different venues across Europe until 2021, when it was planned to take place in Leicester but was cancelled due to the COVID pandemic. It was decided to hold the meeting in Leicester in August 2022 instead, and as an in person meeting rather than an online or hybrid event. Incidentally, the present meeting is the third time the conference has come to the English East Midlands, now in Leicester following previous meetings in the neighbouring cities of Derby and Nottingham.

The sites of previous ECEMs and webpages can be found here..

## Programme of the 21st European Conference on Eye Movements



