# Supplementary material for: Programme of the 21st European Conference on Eye Movements
Source: J Eye Mov Res. 2022 Aug 21;15(5):10.16910/jemr.15.5.1. doi: 10.16910/jemr.15.5.1 (PMC10344597; doi:10.16910/jemr.15.5.1)
Supplement: Supplementary file 1 [file jemr-15-05-a-SD1-01.pdf]

ECEM 2022

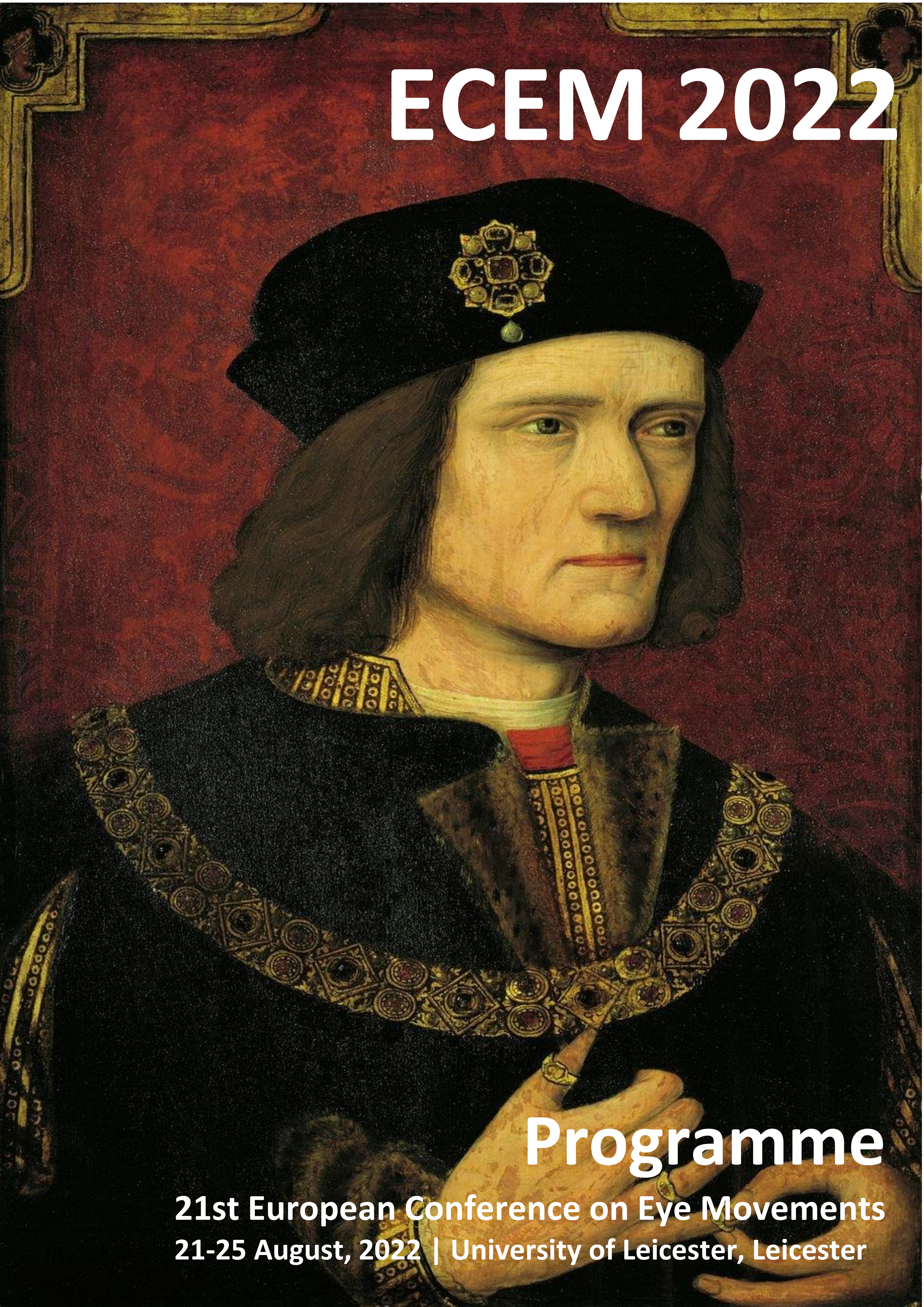

Programme

21st European Conference on Eye Movements

21-25 August, 2022 | University of Leicester, Leicester

Programme of the 21st European Conference on Eye Movements

Edited by Victoria A. McGowan, Ascensión Pagán,

Kevin B. Paterson, and David Souto

August 21st to 25th, 2022

Leicester, UK

About the ECEM Programme Cover Image

The background of the front cover image depicts a portrait of King Richard III of England. King Richard III ruled from 1483-1485 and was the last English king to die in battle, at the battle of Bosworth Field (just outside Leicester). In 2012, an ambitious team from the University of Leicester set out to discover the remains of his body - and found them buried under a car park in Leicester city centre. The discovery and identification of his body is now one of the University’s most celebrated achievements. For more information, see: https://le.ac.uk/richard-iii

The painting is by an unknown artist and is part of the Royal Collection. Examinations of the panel on which the portrait was painted suggests it was created around a date between 1504 and 1520. It also has been suggested that careful examination of the paintings by eye movements alone can reveal some deliberate alteration to the painting, with changes to the right shoulder and coat used to create an unevenness of the shoulders. There is also the suggestion that the eyes have been overpainted to appear greyer, and that the mouth has been changed to turn down at the corners.

These changes most likely reflect propagandist attempts to portray Richard unfavourably. The King had scoliosis of the spine, and the physical difference this produced has been widely used (including by Shakespeare) to signal moral failings and to characterise Richard as an evil man.

For more information, see the Royal Collection Trust

https://www.rct.uk/collection/403436/richard-iii-1452-85

Contents

Foreword	5
About ECEM	6
General Information	7
Practical information	11
Awards	14
The Keith Rayner Memorial Award	14
SR Research Early Career Awards: Best Talk & Best Poster	15
Information for Presenters	16
Keynotes	18
Social Events	21
Brief Programme	23
Full Conference Programme	24
Diagram of Bennett Building	53
Author Index	54
Exhibitors and Sponsors	62
Map of University Campus	66

Foreword

We are very excited to host the 21st European Conference on Eye Movements at the University of Leicester. This conference was originally conceived during the 20th ECEM, held in Alicante in August, 2019. Our plan was to hold the next meeting of this biennial conference in Leicester in August 2021. Little did we know how much the world was about to change.

The COVID virus began circulating in the UK in early 2020, triggering three countrywide lockdowns, with restrictions not fully ending until August 2021. It was around the time of the third lockdown, at the beginning of 2021, that we realised it was unlikely that an in-person ECEM could go ahead in that August. At this point we consulted with Rudolf Groner and the ECEM committee about what we should do. Most academics by this time had experience of attending virtual scientific meetings. However, the decision we took, supported by Rudolf and committee, was that we should not move the conference online, but postpone for a year and plan for an in-person conference in August 2022. This has meant a nervous year of putting plans together while monitoring the news about the outbreak of each new COVID variant. You can imagine our relief as it became clearer that the conference could go ahead in person.

We hope that this will be a memorable ECEM not only because of its disruption by the pandemic. We have an outstanding set of keynotes from different areas of eye movement research, and a cross-disciplinary range of symposia, talks and posters, spanning the Arts and Humanities, visual neuroscience, applied vision research, computer science, experimental psychology, and ophthalmology. The quality of the submissions we have received has been uniformly high and so we hope our ECEM is memorable for the high quality of the eye movement research that is presented.

We also hope you enjoy the location. Leicester is a medium-sized city in the East Midlands of England with a population of around 350K. It has a long history, dating back to pre-Roman times. It is also a highly multicultural city, with more than 70 languages spoken across the city, including Gujarati, Punjabi, Hindi, Bengali, Somali and Urdu. It holds the second-largest Caribbean Carnival in the UK and the largest Diwali Festival outside of India. We hope you can enjoy this diversity during your visit to Leicester.

Finally, we are grateful to everyone who has contributed to the preparation of this meeting: Rudolf Groner, the ECEM committee and especially Ralph Radach, Jukka Hyönä, and Simon Liversedge, our wonderful local administrator Sam Kerr, the International Programme Committee, and our student helpers, with special thanks to our sponsors - SR Research, Tobii Pro, Pupil Labs, iMotions, EyeSeeTec and **Inseye** - without whom this conference would have been difficult to achieve.

The ECEM 2022 Local Organising Committee

About ECEM

ECEM was initiated by Rudolf Groner (Bern), Dieter Heller (Bayreuth at the time) and Henk Breimer (Tilburg) in the 1980s to provide a forum for an interdisciplinary group of scientists interested in eye movements. Since the inaugural meeting in Bern, the conference has been held every two years in different venues across Europe until 2021, when it was planned to take place in Leicester but was cancelled due to the COVID pandemic. It was decided to hold the meeting in Leicester in August 2022 instead, and as an in person meeting rather than an online or hybrid event. Incidentally, the present meeting is the third time the conference has come to the English East Midlands, now in Leicester following previous meetings in the neighbouring cities of Derby and Nottingham.

ECEM 1 1981, September 16-19, Bern, Switzerland
ECEM 2 1983, September 19-23, Nottingham, UK
ECEM 3 1985, September 24-27, Dourdan (Paris), France
ECEM 4 1987, September 21- 24, G Göttingen, Germany
ECEM 5 1989, September 10-13, Pavia, Italy
ECEM 6 1991, September 15-18, Leuven, Belgium
ECEM 7 1993, August 31-September 3, Durham, UK
ECEM 8 1995, September 6-9, Derby, UK
ECEM 9 1997, September 23-26, Ulm, Germany
ECEM 10 1999, September 23-25, Utrecht, the Netherlands
ECEM 11 2001, August22-25, Turku, Finland
ECEM 12 2003, August20-24, Dundee, Scotland
ECEM 13 2005, August14-18, Bern, Switzerland
ECEM 14 2007, August19-23, Potsdam, Germany
ECEM 15 2009, August23-28, Southampton, UK
ECEM 16 2011, August 21-25, Marseille, France
ECEM 17 2013, August11-16, Lund, Sweden
ECEM 18 2015, August 16-21, Vienna, Austria
ECEM 19 2017, August 20-24, Wuppertal, Germany
ECEM 20 2019, August 18-22, Alicante, Spain

General information

Host

ECEM 2022 is hosted by the University of Leicester and the Department of Neuroscience, Psychology and Behaviour, which includes a large interdisciplinary group of experimental psychologists, ophthalmologists, and vision scientists with interests in eye movements.

Local Organising Committee

The conference has been organised by Doug Barrett, Vicky A. McGowan, Ascension Pagan, Kevin B. Paterson, Frank Proudlock, David Souto, and Mervyn G. Thomas.

Support Team

We thank our student support team who are available throughout the conference to answer questions, solve unexpected problems and generally make sure that things run smoothly. During the conference, you can call on the support team for assistance. Support team members will be wearing red ECEM t-shirts. Conference organisers will be wearing black ECEM t-shirts.

Conference Venue

The University was founded as Leicester, Leicestershire and Rutland University College in 1921. The site for the University was donated by a local businessman, Thomas Fielding Johnson, in order to create a living memorial for all local people who made sacrifices during the First World War. This is reflected in the University's motto *Ut vitam habeant* - 'so that they may have life'.

Students were first admitted to the College in 1921, sitting examinations for external degrees awarded by the University of London. In 1927 it became University College, Leicester; 30 years later it was granted its Royal Charter, giving it the status of a University with the right to award its own degrees.

In 2021-22, the University celebrated its centenary.

The conference will be held on the University of Leicester campus. The campus contains a wide range of 20th Century architecture, though the oldest building dates from 1837. The central building, now known as the Fielding Johnson Building, was formerly the Leicestershire and Rutland Lunatic Asylum. The University skyline is punctuated by three distinctive buildings from the 1960s: the Engineering Building, Attenborough Tower and Charles Wilson Building.

The Engineering Building was the first major building by Sir James Stirling. It was completed in 1963 and is notable for the way in which its external form reflects its internal functions. The 18-storey Attenborough Tower has one of the very few remaining paternosters in the UK. The Charles Wilson Building was designed by Sir Denys Lasdun in the brutalist style, and completed in 1963.

The opening ceremony will be held in the Peter Williams Lecture Theatre, followed by a keynote by Iain Gilchrist (sponsored by SR Research). After this, there is an informal welcome event in Fraser Noble Hall, with the opportunity to try some local food (supplied by *Anmol Sweet Centre*), and sample some English regional beer (supplied and served by *The Classroom*) and fine wines.

Keynotes by Fatema Ghasia and Miriam Sperling on Tuesday and Wednesday mornings, respectively, are in the Peter Williams Lecture Theatre. All other talks will take place in the Bennett Building. Keynotes by Ziad Hafed and Monica S. Castelhano on the Monday and Friday, respectively, are in Lecture Theatre 1 (accessible from the ground and lower ground floors). Talks will be held in Lecture Theatre 1, Lecture Theatre 2 (accessible from the lower ground floor), and Lecture Theatre 8 (also accessible from the lower ground floor). We are very pleased to host our sponsors in Lecture Theatre 5 and possibly the atrium area, where they will showcase the latest eye movement recording technology.

Poster sessions on Monday and Tuesday afternoon will be held, with refreshments, in the Square Hall in the Percy Gee Students' Union building.

Registration and Help Desk

We ask that all delegates register on attending the conference. Registration will take place in the foyer to the Peter Williams Lecture Theatre conference prior to the opening ceremony. On all other days, a registration and help desk will be available in the Bennett Building foyer.

We thank SR Research for supplying the registration pack materials, including tote bags, drink holders, and lanyards.

Conference Name Badge

Participants are kindly asked to wear the conference badge at all times during the conference. It entitles them to participate in all activities of the conference. It also makes conversation easier.

Certificate of Attendance

A certificate of Attendance can be provided on request. Please contact the Registration and Help desk.

Online Version of Programme

The most up-to date version of the programme for ECEM 2022 is available at: https://easychair.org/smart-program/ECEM2022/

Wifi Access

The University of Leicester provided free wireless internet access across the campus. This can be accessed in one of two ways.

Visitor WiFi Via Eduroam

You can use the eduroam wifi service if your home institution participates in the eduroam project (http://www.eduroam.org).

Your mobile phone or laptop should connect automatically if you have previously setup eduroam at your home institution, although you may need to enter your username and password, as provided by your home university.

By connecting to eduroam wifi you agree to be bound by the Janet Acceptable Use Policy, the Janet Security Policy, the eduroam(UK) Policy, the eduroam(UK) Data Protection Schedule, the University information security policies and those of your home university.

Visitor WiFi Provided by The Cloud (Sky WiFI)

On your laptop, tablet or phone connect to _The Cloud network

A web browser should open to allow you to register or login (if you have already registered with The Cloud). If a web browser does not appear, open your preferred web browser and navigate to any web page. The Cloud landing page should then open, allowing you to register or login.

Coffee Breaks

Coffee, tea, soft drinks and biscuits will be served in the Bennett Building during official coffee breaks and in the Students' Union during the poster sessions. At other hours, coffee, refreshments and small snacks can be purchased nearby at:

- The Library Café in the **David Wilson Library** – open 8.30 to17.00.
- The **Attenborough Arts Centre** on Lancaster Road – open 9.00 to 16.00.
- Go to Queen's Road (across the Victoria Park) for **Fingerprints**, **Costa Coffee**, **Café Bondade**, and **Northern Cobbler**.
- Go to London Road for the excellent **Six Degrees.**
- Go to King's Street (at end of New Walk) for **Bread and Honey**.

Lunch

A buffet lunch is included in the registration fee and will be provided at the conference venue.

Smoking

Please note that smoking is not permitted inside any buildings, but it is acceptable to smoke outside on campus.

Surroundings

The University of Leicester is located south of Leicester city centre, adjacent to Victoria Park and neighbouring the Highfields and Clarendon Park areas of the city. Leicester railway station is a short 10 minute walk from the campus, at the southern end of London Road.

To get to London Road, walk east along University Road. Where this joins London Road there are numerous cafes, bars and restaurants, including the **Marquis of Wellington** public house (the unofficial post-conference meeting point) and 6 Degrees café. To get to the city centre, you can walk south down London Road, past the railway station, onto Grandby Street. Alternatively, you can take a more picturesque walk along **New Walk**, which is a well-preserved Georgian walkway (dating back to 1869), lined with elegant buildings set behind decorative railings. To get to New Walk, head east from the campus along University Road and turn right onto New Walk about 50 yards (metres) before reaching London Road. New Walk stretches from Victoria Park to King Street in the city centre. If you are attending the conference dinner, this is the ideal route to the City Rooms venue; simply follow New Walk to Kings Street, turn right and cross Belvoir Street onto Market Street, walk to the end of Market Street and cross Horsefair Street onto Hotel Street and City Rooms is on your right.

New Walk used to provide a walkway from the city to Leicester's racecourse, which is now the Victoria Park. The park stretches along north east side of the University, and is an ideal place to take a walk or have a break from the conference. On the far side of Victoria Park is Queen's Road and Clarendon Park, which is a well-conserved area of mostly Victorian era housing as well as cafes, bars and restaurants. Also across the Victoria Park is the Old Horse public house, which is another good venue to meet colleagues for drinks and pub food.

Meeting of ECEM Board

The ECEM board meets on Wednesday, August 24th, 15.00 – 15.30.

Practical information

Important phone numbers

Fire brigade – 999 or 112 for emergency
Police - 999 or 112 for emergency
Ambulance - 999 or 112 for emergency
Medical service – 111 for NHS medical help
Conference emergency phone – please call Kevin Paterson on 07545386642 in an emergency.
General conference enquiries should be made at the Help Desk or via the conference email address (ecem2022@leicester.ac.uk).

COVID-Related Advice

COVID-19 infection remains a risk in the UK. Your welcome bag contains several lateral flow tests and an FP2 face mask. We ask that all attendees test themselves daily and do not attend the conference if they are positive on the test or on symptoms for COVID-19. We also ask that you consider wearing a mask during the conference, to reduce opportunities for infection. If you test positive for COVID-19, please inform us via the conference email address (ecem2022@leicester.ac.uk). This will allow us to monitor your recovery and also the extent of any infection.
Further information about COVID-19 is available from the University and the NHS:

- https://le.ac.uk/living-with-covid-19
- https://www.nhs.uk/conditions/coronavirus-covid-19/

Pharmacy

Nearby pharmacies are:

- City Pharmacy, 110 London Road (tel. 01162545253, opening hours 9.00-13.30, 14.30-15.30)
- Well Pharmacy, 78 Queen’s Road, Clarendon Park (tel. 01162707140, opening hours 9.00-18.00)

Transportation

Much of Leicester is walkable and the city is generally safe. Some care should be taken walking across the Victoria Park after dark.

Bus

Information about local and national bus services is available from Leicester City Council: https://www.leicester.gov.uk/transport-and-streets/travelling-by-bus/

Taxi

Several taxi services operate within the city to local and national airports:

- Everest Taxis Leicester: https://www.everesttaxis.co.uk
- Leicester Black Cabs (operating from Leicester Railway Station: 0116 442 2244
- ABC Taxis (based on London Road): 0116 255 5111
- Hanson Cabs (based on London Road) 0116 233 3333
- An Uber service also operates in Leicester: https://www.uber.com/global/en/cities/leicester/

Cycling

Leicester operates a Santander ebike service with stations around the city: https://rideonleicester.com

Parking

Paid for parking is available near the University at the Victoria Park Car Park : https://www.leicester.gov.uk/transport-and-streets/parking-in-leicester/parking-charges/

Metered on-street parking is available around the University campus and Victoria Park.

NCP also operate a number of car parks across the city: https://www.ncp.co.uk/parking-solutions/cities/leicester/

Tourist information

Leicester is one of the oldest cities in England, dating back to pre-Roman times. It has a long history that we encourage you to explore.

For tourist information, visit https://www.visitleicester.info or the Information Centre at 4A St Martins, Leicester, LE1 5DB

See also a listing for Leicester Museums and Galleries: https://www.leicestermuseums.org

Nearby places to visit include:

- King Richard III Visitor Centre – learn about the discovery of the body of a 500 year old King of England under a car park in Leicester - https://kriii.com
- Leicester Guildhall – The centre of Leicester history for 630 years: https://www.visitleicester.info/see-and-do/leicester-guildhall-p692741

- New Walk Museum and Art Gallery – https://www.leicestermuseums.org/leicester-museum-art-gallery/
- The Golden Mile – an area outside the city centre showcasing the cities many Asian restaurants and shops, and home to the annual Diwali Festival: https://www.visitleicester.info/see-and-do/the-golden-mile-p820881
- Alice Hawkins Statue: - Visit the memorial to a leading suffragette amongst the shoe machinists of Leicester: https://www.visitleicester.info/see-and-do/alice-hawkins-statue-p832161

Awards

The Keith Rayner Memorial Award

It is a pleasure to announce that there will be a Keith Rayner Memorial Award at the Leicester European Conference on Eye Movements 2022 in memory of Keith Rayner and his contribution to the field of eye movements and cognitive psychology. The prize will be awarded to the best student (poster or spoken) presentation at the conference as decided by the adjudication committee. Keith Rayner was a very important figure in the field of eye movements and cognitive psychology. He made a substantial and significant scientific contribution, and he viewed ECEM as a very important conference. Keith was also an amazing mentor to so many young investigators. This award is about honouring both the contribution that Keith made, as well as Keith as a person, and it is hoped that in a small way this award can help continue his legacy. It is also hoped that the award will encourage participation by graduate students in future ECEM conferences.

If you are a student and you are presenting a poster or a paper at ECEM 2022 (i.e., you are the primary author on the presentation and you will deliver the presentation), then you are eligible for consideration for the award. The adjudication process for the award will be as follows: Any person wishing to be considered for the award will be required to send a concise abstract (100 words or less excluding the title), and a statement declaring that the presenter will be a student at the time of the presentation, to the following email address: ECEM2022@leicester.ac.uk. The word limit for the concise abstracts will be strictly observed and any abstract longer than 100 words will not be considered. All abstracts must be submitted to this email address one week before the conference. Any abstracts submitted after this point will not be considered.

All the eligible abstracts will be evaluated by the adjudication committee comprised of well-respected colleagues who will attend the conference. On the basis of the abstracts, members of the adjudication committee will attend candidate presentations for the award, and after the conference a committee decision will be formulated and a winner announced (hopefully at the conference, but if schedule constraints prevent this, then via an announcement on the ECEM website). Thus, decisions will be made according to a two-stage process (shortlist on the basis of concise abstracts, followed by a final decision on the basis of the quality of the shortlisted presentations). The award will take the form of a monetary prize.

If you are eligible for the Keith Rayner Award, and you would like to be considered, I strongly encourage you to submit an abstract. If you have any questions regarding the award, or the adjudication process, please do not hesitate to contact me by email.

With best wishes

Simon P. Liversedge

Early Career Awards: Best Talk & Best Poster Contribution

Two early career (up to 10 years post PhD) awards (a cash prize) will be given for the best talk and for the best poster contribution, sponsored by SR Research.

To be considered for these awards, you need to be the listed as first author of the contribution and to send an email with the abstract for your contribution stating the award to which you would like to be considered to ECEM2022@leicester.ac.uk.

The adjudication process will be similar to the Keith Rayner Memorial Award.

SR Research

EyeLink®

Information for Presenters

Talks

A time slot of 20 minutes is allocated to each talk. Presentations should last 15 minutes followed by 5 minutes of questions and discussion. Computers for PowerPoint or PDF presentations are available in all lecture theatres. If you prepare a PowerPoint presentation, please make sure that it is compatible with Microsoft PowerPoint 2011 for Mac OS or PowerPoint 2010 for Windows. It is recommended to use standard fonts for preparing the presentations to minimize the risk of distorted layout. As a backup, please also bring a PDF version of the presentation. If you intend to present movies, do not only include them in a PowerPoint presentation but also provide the individual movie files.

If you think that your presentation might be susceptible to compatibility issues, you are welcome to check your presentation in the room where it will be given even before the day of your talk. If you need any additional equipment, please let us know in advance by contacting ecem2022@leicester.ac.uk. Please make sure to transfer your presentation files to the computer in the lecture room in the morning or in the break before your session using a USB flash drive. Ask the technical assistants in the lecture rooms for help. They will be present 20 minutes before each session and during the session.

Posters

There will be two poster sessions, on the Monday and Tuesday (15.00-16.30) in the Square Hall in the Percy Gee Students' Union building. Poster presenters are asked to prepare their posters in ISO A0 portrait format (width of 841 mm, and height of 1189mm). We will provide materials for fixing posters to poster boards. Please see the conference programme for a listing of poster presentations in each session. If you are presenting a poster, please take note of the number assigned to your poster in the programme. This number denotes your poster board. Please put your poster on the according poster board during the lunch break of the corresponding day. During the actual poster sessions, the presenting author is expected to be present at the poster. We also recommend either preparing a handout (miniature versions of the poster) or link to an online version of your poster.

Please note that a small number of posters will be displayed without a presenter but with an accompanying QR code. These are cases where it was not possible for the submitter to attend the conference in person, e.g., because of the ongoing conflict situation in Ukraine. In these cases, the poster presenter will be available

online to discuss their poster. Please use the QR code to access their online meeting area.

Chairing

Symposium convenors and session chairs should be present in the lecture theatre at least 10 minutes prior to the start and for the full duration of the chaired session. As a chair, please meet the presenters before their presentations start and make sure that all presenters have uploaded their presentations on the presenting computer prior to the start of the session (at least one helper will provide assistance for the duration of the full chaired session).

Please also make sure that speakers stick to their allotted times. You can use the numbers printed at the end of the programme booklet to notify the presenters of their remaining time (5, 1, and 0 minutes). After the talks, please moderate the discussion while keeping an eye on the start time of the next presenter. Most importantly, take care that the talks start at the precise allocated time according to the published programme. If a talk is cancelled at short notice (e.g. if a speaker simply does not show up), please wait until the official starting time of the next presentation. This will allow possibly interested participants to join the audience in time for each talk. As a consequence, the order of the talks of your chaired session should not be changed as compared to the published programme.

Keynotes

We are excited to have 5 outstanding keynotes for ECEM 2022:

Professor Iain Gilchrist (University of Bristol, UK) is Professor of Neuropsychology at the University of Bristol where he has worked for the last 25 years. He has over 200 publications including papers in Nature, PNAS, Current Biology and Proceeding of the Royal Society. At the heart of this work has been an ongoing fascination with how humans see and a belief in the importance of eye movements in any model of vision. His work has used a range of techniques including psychophysics, MRI, MEG, robotics, neuropsychology, endocrinology and biology. In 2003, he published the influential book Active Vision with John Findlay, and in 2012 along with Simon P. Liversedge and Stefan Everling he edited The Oxford Handbook of Eye Movements.

Professor Ziad Hafed (University of Tübingen, Germany) was trained as an Electrical Engineer at McGill University in Montreal, Canada, where he also obtained his Master's (1999) and PhD (2003) degrees. In 2003, he moved to the Salk Institute for Biological Studies in La Jolla, California as an NSERC (Canada) and Sloan-Swartz (USA) Fellow. Since 2010, Ziad has been leading the Physiology of Active Vision laboratory at the Werner Reichardt Centre for Integrative Neuroscience, an excellence cluster at the University of Tübingen. He is also affiliated with the Hertie Institute for Clinical Brain Research, again in Tübingen. Ziad's interest in biological vision started at the end of his undergraduate studies, during which he worked on computer vision and developed a novel face recognition algorithm for computers. At the time, computer vision was faced with significant challenges to achieving robust performance in tasks that are commonplace nowadays (such as face recognition), and the field as a whole looked for insights from biological vision. Ziad was thus intrigued by understanding human vision, and he embarked on developing biomimetic neural network models of brain circuits in his Master's research, exploring human visual performance in behavioural experiments during his PhD, and investigating neurophysiological mechanisms in awake, behaving primates at the Salk Institute for Biological Studies. He continues to use these approaches in his ongoing research.

Dr Fatema Ghasia (Cole Eye Clinic, USA) is clinician-scientist with expertise in pediatric ophthalmology and binocular vision disorders and research interests in systems neuroscience, with emphasis on human and primate ocular motor control. She is Associate Professor and directs the Vision Neurosciences and Ocular Motility Laboratory at Cole Eye Institute, Cleveland Clinic. Her lab's

primary focus is to understand the role of abnormal neural circuits in strabismus and amblyopia and apply novel strategies for their treatment. As a paediatric ophthalmologist, she has witnessed first-hand the problems and nuances associated with diagnosing and treating patients with binocular vision disorders. As an oculomotor scientist, she has discovered and realized the value of obtaining eye movement recordings in these patients. To resolve a desperate need that she experienced as a clinician, she leveraged her role as an eye movement scientist to understand fixation eye movement abnormalities as they relate to amblyopia diagnosis and treatment outcomes. This has led to the development of cutting-edge infrastructure for tracking eye and head movements simultaneously with high accuracy and precision in children under different viewing conditions. Over the last several years, she has investigated the utility of eye movement measurements in children with binocular vision disorders. The systematic analysis of eye movement traces obtained in the lab has revealed for the first time several features that can be utilized to detect the presence of amblyopia, clinical types, and severity. She has also found that FEM abnormalities correlate with reduced contrast sensitivities and depth perception, and inter-ocular suppression experienced by these patients. Additionally, she has found that assessing FEM characteristics can be a valuable tool to predict functional improvement after patching therapy and recent data as it relates to newer amblyopia dichoptic treatments.

Professor Miriam Spering (University of British Columbia, Canada) is a psychologist and neuroscientist and Associate Professor at the University of British Columbia in Vancouver, Canada, where she also directs the Graduate Program in Neuroscience. She was born and educated in Germany, received her Diploma in Psychology from the University of Heidelberg, and then joined the lab of Karl Gegenfurtner for her PhD in Psychology and Neuroscience at the University of Giessen. After graduating, she completed her postdoctoral training with Marisa Carrasco at New York University before joining UBC in 2010. Her research focuses on the link between visual perception and eye movements and involves healthy adults and various patient populations, including patients with Parkinson’s disease, bipolar disorder and Alzheimer’s disease. Miriam’s group aims at applying eye-movement based experimental paradigms to help us understand disease processes and progression, and to ultimately allow earlier diagnosis. Her research is funded by the Natural Sciences and Engineering Research Council of Canada, and she has received many awards, most recently, a Humboldt Fellowship for a sabbatical in Germany, and a mentorship award of Women in Cognitive Science Canada.

Professor Monica S. Castelhano (Queen’s University, Canada) is a Professor in the Department of Psychology at Queen’s University and is an internationally-renowned scholar examining attentional and memory processes in real-world scenes using eye tracking technology. She received her PhD in 2005 from Michigan State University with John Henderson and joined the lab of the late Keith Rayner as a postdoctoral fellow from 2005-2007 at the University of Massachusetts Amherst. She joined Queen’s University in 2007, where she is the current Chair of the Cognitive Neuroscience research program. For her work, Dr. Castelhano has been awarded an Early Researcher Award from the Ontario Government, and most recently received a prestigious NSERC Accelerator Discovery grant. She was recently appointed a Visiting Professor through the Leverhulme Trust at the University of Central Lancashire with Simon P. Liversedge and Valerie Benson. Her research not only explores attention and memory processing in complex environments, but also investigates the function of these processes underlying clinical populations (e.g. Autism Spectrum Conditions) and how current theory can inform emerging technologies.

Social Events

Opening Ceremony and Welcome Reception

ECEM will formally open from 5pm on Sunday 21st August. We will hold an opening ceremony in the Peter Williams Lecture Theatre, followed by a keynote from Iain Gilchrist. Following the keynote, we invite you to a welcome reception in the Fraser Noble Building from 7pm, where you will have the chance to meet and talk with colleagues, enjoy some local food and taste some regional beer, with wine and soft drinks also available.

Conference Dinner

The conference dinner will be held on the evening of Wednesday 24th August at the City Rooms, which is an elegant Georgian hotel and banqueting hall located on the historic Hotel Street in Leicester city centre. The dinner will be held in the stunning Ballroom and feature a three-course meal inspired by the Indian subcontinent cuisine for which modern Leicester is renowned.

Guests are asked to arrive at 7pm for a drinks reception in the Dining Room. Guests will then be called through to the Ballroom for the conference meal, which will start from around 7.45pm. This is a three-course meal of starters, a main course with rice, bread and salad accompaniments, followed by dessert. Dining is “family-style”, with each course served on the table in sharing dishes. There will be a choice of two meat and two vegetarian/vegan dishes for starters and main course, and a traditional Indian-style sweet for dessert. We will provide dietary information about each course. Wine and water will be included on the table and guests are asked to buy any additional drinks from the downstairs bar. The bar will close at 10pm and the dinner is scheduled to end by 10.30pm.

Table seating will be arranged in advance and name cards will be displayed on each table. Please wear your name badge to the dinner to facilitate conversation.

Social activities on Wednesday 24th August

We have scheduled some excursions and outdoor events for Wednesday afternoon (from 16.30 to 18.30). Possible excursions are a visit to local museums (within a short walk of the University). These include:

- *King Richard III Visitor Centre*: is a museum providing historical background to the life and death of King Richard III, and events and science that led to his discovery in a Leicester carpark.
- *The Guildhall Museum*: Leicester’s oldest surviving medieval building, built around 1390

- *The Magazine* (or Newarke Gateway), which is the last surviving part of the city walls.

Depending on the weather, we plan some outdoor games, including rounders (a British variant of baseball dating back to Tudor times), rugby and football.

We also have liaised with the University Quidditch Club to hold a game of Quidditch. Quidditch is a fast-paced, full-contact sport best described as a mix of rugby, handball and dodgeball. It will be familiar to readers of the Harry Potter novels and those witches and wizards attending ECEM. Notably, the game was first played in the UK officially (outside of Hogwarts) at the University of Leicester in 2012. Full training will be given on the day, although participants are asked to practice their magic in advance. Participation is open to everyone, including muggles.

You may sign up at this link for different events: rb.gy/umfnes

You can also join games on the day by coming to the meeting point on Victoria Park at 16:30 on Wednesday. The meeting point is shown by a red cross on the map on the back of the programme.

Brief Programme

	Sunday	*Monday*	*Tuesday*	*Wednesday*	*Thursday*
8.30-9.30		Registration	Keynote: Fatima Ghasia (Peter Williams LT)	Keynote: Miriam Spering (Peter Williams LT)	COFFEE BREAK
9.30-10.00		COFFEE BREAK			LT1: Symposium to honour Alexander Pollatsek's legacy to eye movement research
10.00-12.00		Parallel sessions: LT1: Higher level I / Social Cognition LT2: Text processing & multi-line reading	Parallel sessions: LT1: Unstable fixation & nystagmus LT2: Eye movement control in reading I LT8: Decision-making	Parallel sessions: LT1: Eye movements in memory processes LT2: Chinese reading LT8: Visuo-motor	
12:00-13:00		LUNCH			
13.00-14.00		Parallel sessions: LT1: Eye-tracking & the Visual Arts LT2: Reading LT8: Clinical & Applied I	Parallel sessions: LT1: Visual search LT2: Reading development LT8: Eye-tracking methods	Parallel sessions: LT2: Special populations LT8: Bilingual reading I	Parallel sessions: LT1: Higher level II LT2: Reading comprehension LT8: Clinical & applied II
14.00-15.00				Exhibitor Events	
15.00-15.30		Poster Session & Coffee Break (Students' Union building)	Poster Session & Coffee Break (Students' Union building)	COFFEE BREAK	
15.30-16.00				Exhibitor Events	Parallel sessions: LT1: Bilingual Reading II LT2: Eye movement control in reading III LT8: Pupillometry
16.00-16.30	Registration (Peter Williams LT)				
16.30-17.00		LT2: Parafoveal processing	Parallel sessions: LT1: Eye movements and higher order text LT2: Eye movement control in reading II LT8: Real world and VR	Social Activities	LT1: Closing Ceremony & Prizes
17.00-17.30	Opening Ceremony (Peter Williams LT)				
17.30-18.30	Keynote: Iain Gilchrist (Peter Williams LT)	Keynote: Ziad Hafed (Bennett LT1)			Keynote: Monica Castelhano (Bennett LT1)
19.00-21.00	Welcome Reception (Fraser Noble Building)				

Full Conference Programme

SUNDAY, AUGUST 21

16.00 – 17.00 REGISTRATION (PETER WILLIAMS LECTURE THEATRE LOBBY)
17.00 – 17.30 OPENING CEREMONY (PETER WILLIAMS LECTURE)
17.30 – 18.30 KEYNOTE: Iain Gilchrist
Integrative Active Vision

19.00 – 21.00 ECEM WELCOME EVENT (FRASER NOBLE HALL)

END OF SUNDAY EVENTS

MONDAY, AUGUST 22

8.30 – 9.30 REGISTRATION (BENNETT BUILDING FOYER)
9.30 – 10.00 COFFEE BREAK (BENNETT BUILDING LECTURE THEATRE 5)

PARALLEL SESSIONS (10.00 – 12.00)

BENNETT LECTURE THEATRE 1. CHAIR: Ignace T. C. Hooge
Thematic session: Higher-level I / Social cognition

10:00 The influence of action affordances and visual salience on viewing of ancient stone tools. *Timothy Hodgson, Maria Silva Gago, Flora Ioannidou and Emiliano Bruner*

10:20 Exploring the mechanisms related to attention biases for threat in social anxiety.
Katerina Pavlou, Athina Manoli, Julie Hadwin and Valerie Benson

10:40 Gaze path category differences lie in early fixation locations
Radha Nila Meghanathan and Stefan Pollmann

11:00 Gaze and speech behavior in parent-child interactions: A dual eye-tracking study
Gijs Holleman, Ignace T. C. Hooge, Jorg Huijding, Maja Deković, Chantal Kemner and Roy S. Hessels

11:20 Looking for interaction? An eye-tracking study on brief social encounters
Roy S. Hessels, Jeroen S. Benjamins, Andrea van Doorn, Jan Koenderink, Gijs Holleman and Ignace T. C. Hooge

11:40 Processing visual information in the classroom – A comparison of teachers' gaze during different didactic activities
Leonie Telgmann and Katharina Müller

BENNETT LECTURE THEATRE 2. Chair: Julie Kirkby
Symposium: Eye movements during Text Processing and Multiline Reading: New Challenges and Opportunities for Insights

10:00 Return sweeps during multiline reading: The influence of text justification and column setting in Chinese (and English) readers.
Jeannie Judge, Mengsi Wang, Donna Gill, Xuejun Bai, Chuanli Zang and Simon P. Liversedge

10:20 The eye-voice span during multiline reading: The implications of return-sweeps
Victoria Adedeji, Julie Kirkby, Martin Vasilev and Timothy Slattery

10:40 Algorithms for assigning fixations to lines of text in multiline passage reading

Jon Carr, Valentina Pescuma and Davide Crepaldi

11:00 Analyzing multi-line reading experiments: Automatized pre-processing and practical recommendations
Sascha Schroeder and Domink Glandorf

11:20 Scanpath regularity as a predictor of performance on reading comprehension assessments
Diane Mézière, Lili Yu, Erik D. Reichle, Titus von der Malsburg and Genevieve McArthur

11:40 Lower-level oculomotor deficits in Schizophrenia during reading: Evidence from return-sweeps
Madison Laks, Andriana Christofalos, Stephanie Wolfer, Elisa C. Dias, Daniel C. Javitt and Heather Sheridan

END OF PARALLEL SESSIONS

12.00 – 13.00 LUNCH BREAK (BENNETT BUILDING)

PARALLEL SESSIONS (13.00 – 15.00)

BENNETT LECTURE THEATRE 1. CHAIR: Anna Miscena

Symposium: Eye-tracking and the Visual Arts

13:00 Is it Art? Effects of framing images as art versus non-art on gaze behaviour and aesthetic judgments
Frank Papenmeier, Gerald Dagit, Christoph Wagner and Stephan Schwan

13:20 Eye-catchers in the museum: Measuring the attraction potential of single artworks
Carola Korhummel, Luise Reitstätter and Raphael Rosenberg

13:40 Eye-tracking and painting restoration
Pablo Fontoura

14:00 The SmART Viewer: the impact of smartphone use on the art viewing experience
Zoya Dare and Raphael Rosenberg

14:20 Two ways of seeing: Investigating the perception of a painting's surface vs of its subject in light of Wollheim's theory of twofoldness.
Anna Miscena and Raphael Rosenberg

BENNETT LECTURE THEATRE 2. Chair: Francoise Vitu

Thematic session: Reading

13:00 Investigating the time-course of visuo-motor and linguistic processes during reading using EEG combined with eye-tracking
Régis Mancini, Laure Spieser, Eric Castet, Boris Burle and Françoise Vitu

13:20 A glimpse into the neural basis for foveal and parafoveal processing: Combined analyses of eye movements and fixation-based fNIRS during reading
Ralph Radach, Andre Rölke, Christian Vorstius and Markus Hofmann

13:40 Contribution of oculometry and EEG synchronization in the understanding of the origin of the dyslexia: Evidence from a phonological lexical decision task in French students
Aikaterini Premeti, Frédéric Isel and Maria Pia Bucci

14:00 Distinct patterns in eye movements and fixation-related potentials put constraints on models of eye movements in reading
Elizabeth Schotter, Sara Milligan, Martín Antúnez and Horacio Barber

14:20 The use of sentential constraint in young and older adults: Evidence from co-registered eye movements and fixation-related potentials
Federica Degno, Ascensión Pagán, Simon P. Liversedge, Richard Kirkden, Sarah J. White and Kevin B. Paterson

14:40 Are there independent effects of constraint and predictability on eye movements during reading?
Roslyn Wong, Aaron Veldre and Sally Andrews

BENNETT LECTURE THEATRE 8. Chair: Trevor Crawford
Thematic session: Clinical and Applied

13:00 Eye-tracker footage is it enough? Retrospective interview with amateur soccer officials using eye tracker footage
Lee Waters, Itay Basvitch and Matthew Timmis

13:20 Expertise effects on fixation locations and durations: Evidence from a music-related visual search task
Nicole Arco, Kinnera Maturi and Heather Sheridan

13:40 A matter of background: How and when does the virtual background in an instructional video Impact Learning?
Andrienne Kerckhoffs, Leen Catrysse and Halszka Jarodzka

14:00 Classification and staging of Parkinson's disease using video-based eye-tracking
Donald Brien, Heidi Riek, Rachel Yep, Jeff Huang, Brian Coe, Corson Areshenkoff, David Breen, David Grimes, Mandar Jog, Donna Kwan, Anthony Lang, Brian Levine, Connie Marras, Mario Masellis, Paula McLaughlin, J. B. Orange, Alicia Peltsch, Angela Roberts, Angela Troyer, Thomas Steeves, Brian Tan, Richard H. Swartz and Douglas Munoz

14:20 Gaze patterns reflect expertise in dynamic echocardiographic imaging
Jochen Laubrock, Alexander Krutz and Sebastian Spethmann

14:40 Pilot study of ocular microtremor in healthy people and in psychopathology
Irina Shoshina, Sergey Lyapunov, Yuliya Simon, Michail Ivanov, Viktoriya Stanovaya, Ivan Lyapunov and Alisa Kosikova

END OF PARALLEL SESSIONS

15.00 – 16.30 POSTER SESSION I (STUDENT UNION SQUARE HALL)

Attention

1. The time course of inhibition of return in an extended saccade sequencing paradigm. *Christof Körner, Margit Höfler, Petra Schönfelder, Helene Halbmayr and Iain D. Gilchrist*
2. Role of Attention in a Dual Task of Localization and Saccadic Remapping. *Anna Dreneva, Uliana Chernova, Maria Ermolova and Joseph MacInnes*
3. A time-course analysis of food cue processing. *Jonas Potthoff and Anne Schienle*
4. Attentional orienting to angry gazes in young children with autism spectrum condition. *Li Zhang, Guoli Yan and Valerie Benson*

Cognition

5. Attentional engagement and disengagement differences for circumscribed interest objects in young Chinese children with Autism Spectrum Condition: An eye movement study. *Li Zhou, Li Zhang, Yuening Xu, Fuyi Yang and Valerie Benson*
6. Altered pupil dynamics associated with cognitive impairment in the progression of Parkinson's disease. *Jeff Huang, Brian Coe, Matthew Smorenburg, Donald Brien, Derek Beaton, Brian Tan, Connie Marras, Jane Lawrence-Dewar, Stephen Strother, Donna Kwan, Paula McLaughlin, Anthony Lang, Sandra Black, Elizabeth Finger, Morris Freedman, Michael Strong, Richard H. Swartz, Carmela Tartaglia, Lorne Zinman, The Ondri Investigators and Douglas Munoz*
7. Study protocol: Eye-tracking parameters as biomarkers of presymptomatic frontotemporal dementia. *Teuni Ten Brink, Lize Jiskoot, Sanne Böing, Stefan Van der Stigchel, Esther van den Berg, Harro Seelaar and Jackie Poos*
8. Eye-tracker procedure to analyze sex differences and strategy induction for solving a mental rotation task. *Raúl Cabestrero, Isabel Orenes, Laura Cepero, Antonio Rodán, Laura Fernández-Méndez, Pedro Montoro, Julia Mayas, Antonio Prieto and Mª José Contreras*
9. Eye-tracking measures of aesthetic experience. *Maartje Raijmakers, Aleksandra Sobolewska and Eftychia Stamkou*
10. Does news source matter? Fake news recognition and message credibility in social media: an eye-tracker approach. *Elena Artemenko, Maksim Terpilovskii, Taisiia Ulianova, Victoria Vziatysheva and Olessia Koltsova*
11. Effects of pictures in instructions for use. *Michael Meng*

12. The effect of bilateral eye movements on episodic memory retrieval: An assessment of ageing and disease effects. *Trevor Crawford and Megan Polden*

Reading

13. Word length, frequency, and predictability effects in eye-movements in L1 reading: A systematic comparison of 12 languages. *Daniil Gnetov, Victor Kuperman and Sascha Schroeder*
14. Investigating the effect of negation on the reading of health statements. *Anna M. Plunkett, Meyrem Tompson, Sarah Wu, Kevin B. Paterson, Sarah J. White and Victoria A. McGowan*
15. Effect of prior knowledge on re-reading behavior after an interruption and text comprehension. *Guillaume Chevet and Véronique Drai-Zerbib*
16. The impact of Inter-word spacing on inference processing: Evidence from eye movements. *Andriana Christofalos, Madison Laks, Nicole Arco and Heather Sheridan*
17. Unexpected sounds inhibit the movement of the eyes during reading and letter scanning. *Michael Lowman, Martin Vasilev, Julie Kirkby, Katherine Bills and Fabrice Parmentier*
18. Eye tracking as a tool for the estimation of a text comprehension. *Konstantin Chernozatonskiy, Victor Anisimov, Ksenia Babanova, Andrey Pikunov, Daria Zhigulskaya and Alexandra Latyshkova*
19. How do we read multimodal advertising posters? *Anastasiia Konovalova and Petrova Tatiana*
20. Effects of auditory distraction during reading: Evidence from the eye movements of young and older adults. *Hayley Barton, Kevin B. Paterson and Victoria A. McGowan*
21. Eye movements during the verification of arithmetic calculations. *Christine Green, Zhichao Zhang, Asha Sajjid, Jonathan Watson, Chuanli Zang, Simon P. Liversedge and Reyhan Furman*
22. A large-scale eye-movement study of reading in Russian children. *Anastasiya Lopukhina, Vladislava Staroverova, Nina Zdorova, Nina Ladinskaya, Anastasiia Kaprielova, Sofya Goldina, Ksenia Bartseva, Olga Vedenina and Olga Dragoy*
23. Parafoveal processing in Chinese reading: Further evidence for the Multi-Constituent Unit (MCU) Hypothesis. *Chuanli Zang, Zijia Lu, Xuejun Bai, Guoli Yan and Simon P. Liversedge*

24. The processing of Chinese three-character idioms with a “1+2” modifier-noun structure. *Ying Fu, Simon P. Liversedge, Xuejun Bai, Guoli Yan and Chuanli Zang*
25. What’s up, popEye? Updates to popEye – an R package to analyse eye movement data from reading experiments. *Sascha Schroeder*
26. Evolution of eye movements across five expertise level during sight reading of music. *Joris Perra and Véronique Drai-Zerbib*
27. Reading speed for different power distributions of progressive power lenses using eye-tracking. *Pablo Concepcion Grande, José Miguel Cleva, Eva Chamorro, Amelia Gonzalez, Paulina Dotor and José Antonio Gomez-Pedrero*

Visuo-Motor

28. The temporal order judgment between saccade and visual stimulation just after saccade. *Junhui Kim and Takako Yoshida*
29. 3D object viewpoint discriminability influences target-selection for saccades. *Emma E.M. Stewart, Ilja Wagner and Roland W. Fleming*
30. Eye am in control: sense of agency for saccades. *Julian Gutzeit, Lynn Huestegge, Jens Kürten and Lisa Weller*
31. Ocular movements to study the influence of defocus induction on VA measurement. *Clara Benedi-Garcia, Pablo Concepcion Grande, José Miguel Cleva, Marta Alvarez and Eva Chamorro*
32. Exploring perceptual decoupling during voluntary and reflexive eye behaviour. *Ziva Korda, Sonja Annerer-Walcher, Christof Körner and Mathias Benedek*
33. Anticipating choice behaviour in strategic settings via machine-learning modeling of scanpath subsequences. *Sean Byrne, Adam Peter Frederick Reynolds, Luca Polonio, Massimo Riccaboni, Carolina Biliotti and Falco Bargagli Stoffi*
34. Interaction of dynamic error signals in saccade adaptation. *Ilja Wagner and Alexander C. Schütz*
35. Do horizontal, vertical and oblique stimulus motion evoke comparable nystagmus and after-nystagmus in human vision? *Kirsten Williams, Paul McGraw, Timothy Ledgeway and Denis Schluppeck*
36. Seeing the Forrest through the trees: Oculomotor metrics are linked to heart rate. *Alex J. Hoogerbrugge, Christoph Strauch, Zoril A. Olah, Edwin S. Dalmaijer, Tanja C. W. Nijboer and Stefan Van der Stigchel*
37. Cyclovergence movements in presence of vertical shear disparity across depth planes. *Saad Almajed, Philip Duke and David Souto*

38. The effects of personal interest level on gaze bias for visual preference decisions. *Anna Yoshida and Keiko Momose*
39. It's hard not to look - but possible: Using eye movements to study inhibitory control difficulties in multiple-action control. *Jens Kürten, Tim Raettig, Julian Gutzeit and Lynn Huestegge*
40. Relating asthenopic symptoms to optometric measures and parameters of binocular vision. *Joëlle Joss and Stephanie Jainta*

BENNETT LECTURE THEATRE 2. Chair: Ralph Radach

Thematic session: Parafoveal Processing

16:30 New evidence on parafoveal syntactic processing during reading
Laura Schwalm and Ralph Radach

16:50 Transposed-Letter allographic effects in Arabic: Evidence from the boundary technique
Maryam A. Aljassmi, Sami Boudelaa, Kevin B. Paterson and Manuel Perea

17:10 Relating foveal and parafoveal processing efficiency with word-level eye-movement measures of text reading
Timo Heikkilä and Jukka Hyönä

17.30 – 18.30 LECTURE THEATRE 1
KEYNOTE: Ziad Hafed
A vision for orienting in primate oculomotor control circuitry

END OF MONDAY EVENTS

TUESDAY, AUGUST 23

8.30 – 9.30 PETER WILLIAMS LECTURE THEATRE
KEYNOTE: Fatema Ghasia
Miniscule eye movements play a major role in binocular vision disorders

9.30 – 10.00 COFFEE BREAK (BENNETT BUILDING LECTURE THEATRE 5)

PARALLEL SESSIONS (10.00 – 12.00)

BENNETT LECTURE THEATRE 1. CHAIR: Frank Proudlock
Symposium: Unstable Fixation and Nystagmus with a Focus on the Next Generation of Researchers

10:00 Fixation eye movements in pediatric eye diseases
Fatema Ghasia

10:20 Accuracy and precision of fixation is correlated with gaze angle
Onyeka Amiebenomo, Lee McIlreavy, Jonathan Erichsen and J Margaret Woodhouse

10:40 Investigating "Time to See" in infantile nystagmus
Katherine Ward, Fergal Ennis, Lee McIlreavy, Matt J. Dunn and Jonathan T Erichsen

11:00 Phenotyping in infantile nystagmus
Helen Kuht and Mervyn G. Thomas

11:20 The fovea is horizontally elongated in infantile nystagmus
Nikita Thomas, Jennifer H. Acton, Jonathan T. Erichsen, James Fergusson and Matt J. Dunn

11:40 Abnormal electroretinography in albinism and idiopathic infantile nystagmus
Zhanhan Tu, Christopher Degg, Michael Bach, Rebecca McLean, Viral Sheth, Mervyn G. Thomas, Irene Gottlob and Frank Proudlock

BENNETT LECTURE THEATRE 2. Chair: Elizabeth Schotter
Thematic session: Eye Movement Control in Reading I

10:00 Understanding the visual constraints on lexical processing: New empirical and simulation results *Aaron Veldre, Erik D. Reichle, Lili Yu and Sally Andrews*

10:20 Theorizing dynamic adjustment of saccade lengths in reading and dual-stage progression of visual word recognition
Jarkko Hautala

10:40 Print size as an explanation for inter-language differences in eye-movement behaviour during reading: Empirical and neurocomputational evidence
Françoise Vitu, Hossein Adeli and Gregory J. Zelinsky
11:00 Eye movement control during reading and skimming: Effects of word length
Shihui Wu, Kayleigh L. Warrington, Erik D. Reichle, Kevin B. Paterson and Sarah J. White
11:20 A cross-linguistic study of spatial parameters of eye-movement control during reading
Victor Kuperman
11:40 Individual Differences and the Impact of Word Frequency on Eye Movements during Reading
Charlotte Lee, Hayward Godwin, Hazel Blythe and Denis Drieghe

BENNETT LECTURE THEATRE 8. Chair: Miriam Spering
Thematic session: Decision-making
10:00 The contribution of visual conduction delay to saccadic reaction time
Aline Bompas, Craig Hedge and Petroc Sumner
10:20 Uncertainty driven gaze selection
Daniela Pamplona and Antoine Manzanera
10:40 Motivation by reward increases performance beyond the speed-accuracy trade-off by improving distractor suppression
Christian Wolf and Markus Lappe
11:00 Decision making, reward and eye movements
Eugene McSorley and Rachel McCloy
11:20 What drives pupil dilation during decision making– surpise or uncertainty?
Péter Pajkossy and Gábor Gesztesi
11:40 Error inconsistency does not generally inhibit saccadic adaptation
Thomas Eggert, Katharina Kaltenbach and Andreas Straube

END OF PARALLEL SESSIONS

12.00 – 13.00 LUNCH BREAK (BENNETT BUILDING)

PARALLEL SESSIONS (13.00 – 15.00)

BENNETT LECTURE THEATRE 1. CHAIR: Amelia Hunt

Thematic session: Visual Search

13:00 Age-related changes in oculomotor indices of top-down selection during visual search
Fengjun Zhang, Hongyu Xie, Jingxin Wang and Doug Barrett

13:20 Efficient eye movements during search for an object, inefficient eye movements during search for a feature
Anna Nowakowska, Alasdair Clarke, Josephine Reuther and Amelia Hunt

13:40 Categories of eye movement errors and their relationship to strategy and performance
Letizia Caruso, Anna Nowakowska, Alasdair Clarke, Amy Irwin and Amelia R. Hunt

14:00 Does pre-crastination explain why some observers make sub-optimal eye movements in a visual search task
Alasdair Clarke, Kyle Sauerberger, Anna Nowakowska, David Rosenbaum, Thomas Zentall and Amelia Hunt

14:20 Developing a collaborative framework for naturalistic visual search.
Charli Sherman, Anna Hughes and Alasdair Clarke

BENNETT LECTURE THEATRE 2. Chair: Hazel Blythe

Thematic session: Reading Development

13:00 Seven Years Later – Executive functioning predicts the development of the perceptual span during reading
Johannes M Meixner and Jochen Laubrock

13:20 The importance of the first letter in children's parafoveal pre-processing in English: Is it phonologically or orthographically driven?
Sara Milledge, Simon P. Liversedge and Hazel Blythe

13:40 The effect of relevance in children's reading of science texts
Tuomo Häikiö, Oksana Kanerva, Johanna Kaakinen and Mirjamaija Mikkilä-Erdmann

14:00 Children's processing of written irony: An eye-tracking study
Henri Olkoniemi, Sohvi Halonen, Penny Pexman and Tuomo Häikiö

14:20 Concurrent and predictive validity of reading assessment by eye tracking and machine learning
Gustaf Ö. Seimyr, Elizabeth B. Meisinger, Hannah Manning and Mattias Nilsson

14:40 The eye-voice span in children: Exploring individual differences
Julie Kirkby, Victoria Adedeji, Martin Vasilev and Timothy Slattery

BENNETT LECTURE THEATRE 8. Chair: Roy S. Hessels

Thematic session: Eye-tracking methods

13:00 Fixation classification: how to merge and select fixation candidates
Ignace Hooge, Diederick Niehorster, Marcus Nyström, Richard Andersson and Roy S. Hessels

13:20 Web-based attention-tracking with an eye-tracking analogue is reliable and valid
Edwin S. Dalmaijer, Alexander L. Anwyl-Irvine and Thomas Armstrong

13:40 Characterising eye movement events with an unsupervised hidden Markov model
Ingmar Visser, Šimon Kucharský and Malte Lüken

14:00 The amplitude of small eye movements can be accurately estimated with video-based eye trackers
Marcus Nyström, Diederick C. Niehorster, Richard Andersson, Roy S. Hessels and Ignace T. C. Hooge

14:20 Event level evaluation of eye movement event detectors
Raimondas Zemblys and Mikhail Startsev

14:40 Eye tracking: empirical foundations for a minimal reporting guideline
Roy S. Hessels, Kenneth Holmqvist, Saga Lee Örbom, Ignace T. C. Hooge, Diederick C. Niehorster, Robert G. Alexander, Richard Andersson, Jeroen S. Benjamins, Pieter Blignaut, Anne-Marie Brouwer, Lewis L. Chuang, Kirsten A. Dalrympleke, Denis Drieghe, Matt J. Dunn, Ulrich Ettinger, Susann Fiedler, Tom Foulsham, Jos N. van der Geest, Dan Witzner Hansen, Samuel B. Hutton, Enkelejda Kasneci, Alan Kingstone, Paul C. Knox, Ellen M. Kok, Helena Lee, Joy Yeonjoo Lee, Jukka M. Leppänen, Stephen Macknik, Päivi Majaranta, Susana Martinez-Conde, Antje Nuthmann, Marcus Nyström, Jacob L. Orquin, Jorge Otero-Millan, Soon Young Park, Stanislav Popelka, Frank Proudlock, Frank Renkewitz, Austin Roorda, Michael Schulte-Mecklenbeck, Bonita Sharif, Frederick Shic, Mark Shovman, Mervyn G. Thomas, Ward Venrooij, and Raimondas Zemblys

END OF PARALLEL SESSIONS

15.00– 16.30 POSTER SESSION II (STUDENT UNION SQUARE HALL)

Attention

1. Seeing your own webcam image feels distracting, but does not hurt learning: A webcam-based eyetracking study. *Gesa van den Broek, Vincent Hoogerheide and Ellen Kok*
2. Attentional biases in the size of fixational saccades. *Baiwei Liu, Anne Zonneveld and Freek van Ede*
3. Eye movements in three-dimensional multiple object tracking. *Qi Zhang, Juntian Lin, Man Jia, Jie Li and Yu Zhang*
4. Modeling task-dependency of eye movement during scene viewing. *Lisa Schwetlick, Daniel Backhaus and Ralf Engbert*
5. Using Eye tracking techniques for oculomotor sign of neglect. *Marina Shurupova, Kseniya Lizunkova, Alina Aizenshtein, Anna Dreneva and Alexander Latanov*
6. Oculomotor control and dual-task Interference. *Aleks Pieczykolan and Lynn Huestegge*

Memory

7. Testing memory strength with pupil dilation as a function of strategic and automatic memory retrieval. *Ádám Albi and Péter Pajkossy*
8. Eye-tracking in innovative neuropsychological assessment of visual working memory. *Sanne Böing, Teuni Ten Brink, Alex Hoogerbrugge, Tanja Nijboer and Stefan Van der Stigchel*
9. Pupil responses: indices of individual memory performance. *Gábor László Bényei and Péter Pajkossy*
10. The when and where of the Looking at Nothing Effect: Examining eye movements during memory retrieval. *Ruhi Bhanap, Klaus Oberauer and Agnes Rosner*
11. The context effect on implicit sequence learning using an ocular version of the Serial Reaction Time (O-SRT) task. *Eli Vakil and Simone Schwizer Ashkenazi*

Methods

12. eyetRack - Shiny application for recurrence quantification analysis. *Veronika Kalabusova, Kamila Facevicova and Stanislav Popelka*

13. An open-source device for vestibular stimulation and eye-movement tracking in head-fixed mice. *Alexandra Tran Van Minh, Xavier Cano-Ferrer and Ede Rancz*
14. Yarbus in the age of Webcam Eye-tracking. *Divya Seernani, Amandine Grappe, Andrew Korepanov, Kåre Jensen, Kerstin Wolf, Jessica Wilson and Nadia Pedersen*
15. GlassesValidator: Data quality tool for eye tracking glasses. *Diederick C. Niehorster, Roy S. Hessels, Jeroen S. Benjamins, Marcus Nyström and Ignace T. C. Hooge*
16. A field test of appearance-based gaze estimation. *Niilo Valtakari, Roy S. Hessels, Diederick C. Niehorster, Chantal Kemner and Ignace T. C. Hooge*

Reading

17. Interactive effects of semantic diversity and word frequency in natural reading. *Neslihan Caliskan, Sara Milligan, Milca Jaime Brunet and Elizabeth Schotter*
18. Individual differences in word learning associated with reading skill and vocabulary: An eye-movement investigation. *Emily J. Bellerby, Sara Milledge, Kristofor McCarty and Hazel Blythe*
19. GECO-CN: Ghent Eye-Tracking COrpus of sentence reading for Chinese-English bilinguals. *Longjiao Sui*
20. The role of phonological and orthographic parafoveal processing during silent reading in Russian children and adults. *Vladislava Staroverova, Anastasiya Lopukhina, Nina Zdorova, Nina Ladinskaya, Olga Vedenina, Sofya Goldina, Anastasiia Kaprielova, Ksenia Bartseva and Olga Dragoy*
21. Reading search page results: Evidence from an eye tracking study on 11-12-year-olds. *Oksana Kanerva, Tuomo Häikiö, Johanna K. Kaakinen and Mirjamaija Mikkilä-Erdmann*
22. Beginning to characterise children’s eye movement control during reading in English: A corpus study. *Sara Milledge, Chuanli Zang, Hazel Blythe and Simon P. Liversedge*
23. The processing strategies for illustrated science reading and Chinese academic words with different semantic transparency among middle-school students: An eye-tracking study. *Pin-Hsien Kuo and Yu-Cin Jian*
24. Do Chinese deaf readers develop a unique cognitive mechanism during visual word recognition? The effect of oral language experience and reading ability. *Zebo Lan, Nina Liu, Xiaoyuan Yuan, Meihua Guo, Guoli Yan and Valerie Benson*

25. Eye movements and reading in children who survived cerebellar tumors. *Sofia Mironets, Marina Shurupova and Anna Dreneva*
26. Scanpath analysis of eye movements during reading in children with high risk of dyslexia. *Anastasiya Lopukhina, Olga Parshina, Sofya Goldina, Ekaterina Iskra, Margarita Serebryakova, Vladislava Staroverova, Nina Zdorova and Olga Dragoy*

L2 Reading

27. Metacognitive modeling effect of reading illustration first for EFL readers: A study of eye movement evidence. *Ming-Yi Hsieh and Sunny S. J. Lin*
28. Lexical access in L2 reading: evidence from self-paced reading and eye tracking data. *Daria Chernova, Marina Norkina and Svetlana Alexeeva*
29. The role of the left perceptual span in L2 reading: An eye-tracking study. *Agnesa Xheladini, Leigh Fernandez and Shanley Allen*
30. Silent or oral reading in L2: An Eye-tracking study. *Tatiana Petrova and Michael Ivanov*

Real-World

31. A two-tier taxonomy of gaze behaviours for free-moving participants. *Mark Shovman, Anna Morozov and Ksenia Burgart*
32. Fixation sequences when walking up and down stairs in daily life. *Andrea Ghiani, Joost G. Driessen, Liz R. Van Hout and Eli Brenner*
33. Investigating the effects of task and body movement on the generalizability of scene viewing experiments. *Daniel Backhaus and Ralf Engbert*
34. Automated discrimination of stable and non-stable gaze events in dynamic natural conditions. *Ashkan Nejad, Eva Postuma, Gera de Haan, Joost Heutink and Frans Cornelissen*

Social Cognition

35. Investigating face perception during free-viewing in a naturalistic virtual environment. *Debora Nolte, Marc Vidal De Palol and Peter König*
36. Looking for speaking: What determines language-specific expressions in motion event descriptions. *Yuko Yoshinari*
37. Gaze aversions serve as social signals conveying the performer’s cognitive state. *Amit Zehngut, Dekel Abeles and Shlomit Yuval-Greenberg*
38. Gaze aversion in human-robot Interaction: Case studies in physical and virtual settings. *Cengiz Acarturk, Bartlomiej Sniezynski, Bipin Indurkhya, Piotr Nawrocki and Sinan Kalkan*

39. Semantics of gaze: Deciphering the meaning of a listener’s gaze direction, gaze position changes, and blink frequency. *Lynn Huestegge, Eva Landmann, Christina Breil and Anne Böckler-Raettig*

PARALLEL SESSIONS (16.30 – 18.30)

BENNETT LECTURE THEATRE 1. Chair: Mesian Tilmatine

Symposium: Eye Movements as a Measure of Higher-level Text Processing

16:30 Mind-wandering during reading of Siri Hustvedt's Memories from the Future: Evidence from eye tracking.
Johanna K. Kaakinen, Emilia Ranta and Jaana Simola

16:50 Reading Russian poetry: An expert–novice study
Danil Fokin and Stefan Blohm

17:10 Unravelling the social-cognitive potential of narratives using eye-tracking
Lynn Eekhof, Kobie van Krieken, José Sanders and Roel Willems

17:30 Different Kinds of Simulation During Literary Reading: Insights from a Combined fMRI and Eye Tracking Study
Marloes Mak, Myrthe Faber and Roel Willems

17:50 Effects of centrality on eye movements: Predictions by computational language models
Sascha Schroeder

18:10 Eye movements as a measure of immersion and foregrounding in narrative poetry reading
Mesian Tilmatine

BENNETT LECTURE THEATRE 2. Chair: Denis Drieghe

Thematic session: Eye Movement Control in Reading II

16:30 Word difficulty determines regression accuracy in sentence reading
Anne Friede, Christian Vorstius, Albrecht Inhoff and Ralph Radach

16:50 Does visual-similarity cause more regressions in reading? An eye-tracking based study.
Mingqing Xie, Natalie Tze and Patrick Sturt

17:10 When functions words carry content
Joao Vieira, Elisangela Teixeira, Erica Rodrigues and Denis Drieghe

17:30 Does omitting mandatory commas affect the reading process?
Bernhard Angele, Ismael Gutiérrez Cordero, Manuel Perea and Ana Marcet

17:50 TheroleofspacesinreadingFinnishText
Raymond Bertram

18:10 The role of visual crowding in eye movements during reading: Effects of text spacing
Tzu-Yao Chiu and Denis Drieghe

BENNETT LECTURE THEATRE 8. Chair: Tom Foulsham

Thematic session: Real World and Virtual Reality

16:30 Characterization of naturalistic free viewing behaviour across the lifespan
Rachel Yep, Brian White, Heidi Riek, Olivia Calancie, Ryan Kirkpatrick, Julia Perkins, Matthew Smorenburg, Donald Brien, Brian Coe, Laurent Itti and Douglas Munoz

16:50 Visual stability in naturalistic scenes
Jessica Parker and Caglar Tas

17:10 Finding landmarks – An investigation of viewing behaviour during spatial navigation in VR using a graph-theoretical analysis approach
Jasmin L. Walter, Lucas Essmann, Sabine König and Peter König

17:30 An online experiment with deep learning models for tracking eye movements via webcam
Shreshth Saxena, Lauren K. Fink and Elke B. Lange

17:50 Georeferencing of eye movement data using ET2Spatial software
Stanislav Popelka, Minha Noor Sultan and Josef Strobl

END OF PARALLEL SESSIONS

END OF TUESDAY EVENTS

WEDNESDAY, AUGUST 24

8.30 – 9.30 PETER WILLIAMS LECTURE THEATRE
KEYNOTE: Miriam Spering
Eye Movements as a Window into Human Decision-Making

9.30 – 10.00 COFFEE BREAK (BENNETT BUILDING LECTURE THEATRE 5)

PARALLEL SESSIONS (10.00 – 12.00)

BENNETT LECTURE THEATRE 1. Chair: Shlomit Yuval-Greenberg
Symposium: The role of Eye Movements in Memory Processes: Between Working Memory and Long-term Memory

10:00 Utilising directional microsaccade biases as a 'tool' to track selective attention inside working memory in time and space
Freek van Ede

10:20 What the variations in saccade metrics and visual memory across the visual field tell about saccadic selection in visual working memory
Sven Ohl

10:40 Eye movements as a window into time-dependent memory processes
Miriam Spering, Annick Langlois, Philipp Kreyenmeier, Lisa Kroell, Anna Heuer and Martin Rolfs

11:00 Gaze behaviour supports episodic memory: insights from electrophysiological data
Mikael Johansson, Inês Bramão, Andrey Nikolaev and Roger Johansson

11:20 The intersection of memory and active vision in aging
Jennifer Ryan, Jordana Wynn, Kelly Shen and Zhong Xu Liu

11:40 What makes eye movements a memory retrieval cue?
Shlomit Yuval-Greenberg and Keren Taub

BENNETT LECTURE THEATRE 2. Chair: Simon P. Liversedge
Thematic session: Chinese Reading

10:00 Word length and frequency in Chinese reading: Evidence from eye movements
Ying Fu, Simon P. Liversedge, Maleeha Moosa, Xuejun Bai and Chuanli Zang

10:20 The role of radicals during parafoveal processing of Chinese characters
Federica Degno, Lixin Wei, Simon P. Liversedge, Manman Zhang, Mengsi Wang and Chuanli Zang

10:40 Reading Classical Chinese fables with implicit moral point: Eye-movement evidences of lexical difficulty, paragraph focus and order effects
Zheng-Hong Guan and Sunny S. J. Lin

11:00 Foveal and parafoveal processing of Chinese four-character idioms and phrases in reading
Chuanli Zang, Shuangshuang Wang, Xuejun Bai, Guoli Yan and Simon P. Liversedge

11:20 Flexible parafoveal encoding of character order supports word predictability effects in Chinese for both young and older adult readers
Min Chang, Kuo Zhang, Lisha Hao, Kevin B. Paterson, Kayleigh L. Warrington and Jingxin Wang

11:40 Word length effect in developing Chinese readers during sentence reading
Nina Liu, Xiyuan Li, Yongsheng Wang, Guoli Yan, Ascensión Pagán and Kevin B. Paterson

BENNETT LECTURE THEATRE 8. Chair: Ziad Hafed
Thematic session: Visuo-motor

10:00 Neglect-like visual exploration by gaze-contingent manipulation of scenes
Andreas Sprenger, Lisa Kunkel Genannt Bode, Christoph Helmchen and Bjoern Machner

10:20 Familiar objects benefit more from transsaccadic feature predictions
Nedim Goktepe and Alexander C. Schütz

10:40 This vortex cannot be pursued
Krischan Koerfer, Tamara Watson and Markus Lappe

11:00 Nasal-temporal differences in the Remote Distractor Effect: how the presence of placeholders affects saccade latencies
Soazig Casteau, Robin Walker and Daniel Smith

11:20 Neural correlates of handedness related modulation of the Vestibular-Ocular Reflex
Qadeer Arshad, Angela Bonsu, Mishaal Sharif, Giuseppe Gava and Adolfo Bronstein

11:40 Sound influences visually-guided eye and hand movements during manual interception
Anna Schroeger, Philipp Kreyenmeier, Markus Raab, Rouwen Cañal-Bruland and Miriam Spering

END OF PARALLEL SESSIONS

12.00 – 13.00 LUNCH BREAK (BENNETT BUILDING)

PARALLEL SESSIONS (13.00 – 15.00)

BENNETT LECTURE THEATRE 2. Chair: Valerie Benson

Thematic session: Special Populations

13:00 Activation of ASL signs during sentence reading for deaf readers: evidence from eye-tracking
Emily Saunders, Jonathan Mirault and Karen Emmorey

13:20 Skilled, efficient reading in deaf child signers: A small-scale eye-tracking study
Frances Cooley and Elizabeth Schotter

13:40 Gender and the formation of co-reference links during reading in autism
Philippa Howard, Valerie Benson and Simon P. Liversedge

BENNETT LECTURE THEATRE 8. Chair: Ascensión Pagán

Thematic session: Bilingual Reading

13:00 Bilingual parafoveal processing during reading: Orthographic preview benefits in L1 and L2
Simon Tiffin-Richards and Ascensión Pagán

13:20 Semantic and orthographic parafoveal processing in bilingual readers
Leigh Fernandez, Christoph Scheepers and Shanley Allen

13:40 Your eyes tell your story: how eye-movement patterns during natural reading develop with L2 proficiency
Shiyu He, Dagmar Divjak and Petar Milin

END OF PARALLEL SESSIONS

14.00 – 15.00 SPONSORED EVENTS BY SR RESEARCH AND TOBII PRO
BENNETT LECTURE THEATRE 1. SR-Research

Measuring and Maximizing Eye Tracker Data Quality: Understanding the key determinants of eye tracking data quality is critical for researchers who want to maximize their ability to detect significant effects in gaze metrics and generate and report high quality, replicable data. We will outline the key determinants of eye tracking data quality, and provide suggestions for how key data quality metrics such as accuracy and precision can be derived from EyeLink data. We will also provide some very useful tips and tricks that you can use to maximize your eye tracking data quality – from optimizing camera and participant setup, to choosing the most appropriate calibration model.

BENNETT LECTURE THEATRE 2. Tobii Pro

Welcome note by Dr. Camilla Fahlström and Dr. Peter Reuter

Ieva Miseviciute will provide an overview of the new eye tracking signal developed at Tobii - eye openness, which provides the basis for accurate eyelid movement detection, including blinks. Ieva will discuss the key benefits eye openness signal brings to eye tracking research.

Pierre Karlsson will share results of a large field test report for Tobii Pro Spectrum and Tobii Pro Fusion. This methodology is used to understand eye tracker performance in less constrained conditions, as well as how participant characteristics influence eye tracking data.

Antoine Luu will present different eye tracking applications such as reading studies, vision assessment, and VR/XR, as well as technical and business solutions offered to researchers to take their research into the commercialization steps.

Andreu Oliver Moreno will introduce our newly released Funding Support Services and highlight how we can help you secure funding for your next project with eye tracking.

15.00 – 15.30 TEA / COFFEE BREAK (BENNETT BUILDING)

14.00 – 15.00 SPONSORED EVENTS BY SR RESEARCH AND TOBII PRO

BENNETT LECTURE THEATRE 1. SR-Research

Recent Software Developments: We will outline recent updates to our software, including the updated API with new example templates for integration with common stimulus presentation solutions such as psychtoolbox, PsychoPy, E-Prime and more. We'll also highlight some very neat new features in Data Viewer, Experiment Builder and WebLink.

BENNETT LECTURE THEATRE 2. Tobii Pro
Continuation of the above.

16.30 – 18.30 EXCURSIONS AND OUTSIDE EVENTS
19.00 – 22.30 CONFERENCE DINNER (CITY ROOM, HOTEL STREET)

END OF WEDNESDAY EVENTS

THURSDAY, AUGUST 25

9.00 – 9.30 COFFEE (BENNETT BUILDING LECTURE THEATRE 5)

BENNETT LECTURE THEATRE 1. Chair: Jukka Hyönä

Symposium to Honour Alexander Pollatsek's Legacy to Eye Movement Research

9:30 Reading compound words in Finnish and Chinese: An eye-tracking study
Jukka Hyönä, Lei Cui, Birgitta Paranko and Timo Heikkilä

09:50 Scene perception through time and space
Monica S. Castelhano

10:10 Words, letters, and the front-end of word identification and reading
Manuel Perea and Ana Marcet

10:30 A multiverse exploration of choices in cleaning and analysing eye movements during reading
Denis Drieghe, Charlotte Lee and Hayward Godwin

10:50 Operationalisation of processes over linguistic units in reading: Cross-linguistic, acuity and lexical processing considerations
Simon P. Liversedge

11:10 Sandy Pollatsek's legacy to visual cognition (pre-recorded presentation)
John Henderson

11:25 Eye glance behaviors: Their role in theory and practice (pre-recorded presentation)
Donald Fisher

11:40 Exploring mechanisms of Chinese reading with Sandy Pollatsek (pre-recorded presentation)
Xingshan Li

12.00 – 13.00 LUNCH BREAK (BENNETT BUILDING)

PARALLEL SESSIONS (13.00 – 15.00)

BENNETT LECTURE THEATRE 1. Chair: Yoni Pertzov

Thematic session: Higher-level II

13:00 Search for the Unknown: Guidance of Visual Search in the Absence of an Active Template
Oryah Lancry-Dayan and Yoni Pertzov

13:20 Attenuation of visual exploration due to accessing of internally stored representations
Tal Nahari, Eran Eldar and Yoni Pertzov

13:40 Investigating the role of Theory of Mind on the processing of dramatic irony scenes in film
Cynthia Cabañas, Atsushi Senju and Tim J. Smith

14:00 Mental detection using eye movements. ~ Eyes tell you the mental status
Ayumi Takemoto, Inese Aispuriete, Laima Niedra and Lana Franceska Dreimane

BENNETT LECTURE THEATRE 2. Chair: Sascha Schroeder

Thematic session: Reading Comprehension

13:00 How do we resume our reading after an interruption? Effects of interruption on eye movements and reading comprehension
Véronique Drai-Zerbib and Chevet Guillaume

13:20 Effects of “desired difficulty” on eye movements and comprehension in reading
Christian Vorstius, Laura Schwalm and Ralph Radach

13:40 The role of context in the processing of semantic ambiguities: Eye-tracking evidence from younger and older adults
Tami Kalsi, Kevin B. Paterson and Ruth Filik

14:00 Effects of reading goals on processing of syntactic ambiguity, semantic plausibility and sentence wrap-up: Insights from eye movement behaviour
Fawziah S. Qahtani, Kayleigh L. Warrington, Kevin B. Paterson and Sarah J. White

14:20 How does word order influence natural reading?
Petar Atanasov, Simon P. Liversedge and Federica Degno

14:40 How early do readers extract the meaning of an emoji?: Evidence from eye movements
Heather Sheridan, Andriana Chistofalos, Eliza Barach and Laurie B. Feldman

BENNETT LECTURE THEATRE 8. Chair: Zhanhan Tu

Thematic session: Clinical and Applied II

13:00 Active vision in sight recovery individuals with a history of long-lasting congenital visual deprivation
José P. Ossandón, Paul Zerr, Idris Shareef, Ramesh Kekunnaya and Brigitte Röder

13:20 Environmental demand influences scanning behaviour in people with hemianopia
Eva Postuma, Ashkan Nejad, Gera de Haan, Joost Heutink and Frans Cornelissen

13:40 Body (dis)satisfaction in transgender and cisgender people: A novel eye-tracking study to explore attentional bias
Filipe Cristino and Beth A. Jones

14:00 Saccadic temporal prediction in typically developing youth and in psychiatric adolescents with impulsivity
Olivia Calancie, Donald Brien, Jeff Huang, Brian Coe, Linda Booij, Sarosh Khalid-Khan and Douglas Munoz

14:20 Pro- and anti-saccade parameters reveal discrete neural processes and differentially associate with cognitive domains in neurodegenerative disease
Heidi C. Riek, Brian Coe, Donald Brien, Jeff Huang, Agessandro Abrahao, Stephen Arnott, Derek Beaton, Malcolm Binns, Sandra Black, Elizabeth Finger, Morris Freedman, Donna Kwan, Anthony Lang, Brian Levine, Wendy Lou, Connie Marras, Mario Masellis, Paula McLaughlin, J. B. Orange, Angela Roberts, Stephen Strother, Kelly Sunderland, Richard H. Swartz, Brian Tan, Carmela Tartaglia, Angela Troyer, Lorne Zinman, The Ondri Investigators and Douglas Munoz

14:40 Utility of eye tracking in visual cortical prostheses – preliminary patient testing results
Avi Caspi

END OF PARALLEL SESSIONS

15.00 -15.30 TEA / COFFEE BREAK (BENNETT BUILDING)

PARALLEL SESSIONS (15.30 – 16.30)

BENNETT LECTURE THEATRE 1. Chair: Ascensión Pagán
Thematic session: Bilingual Reading II

15:30 Evaluating the vocabulary coping strategies of L2 readers through eye tracking
Caleb Prichard and Andrew Atkins

15:50 Processing and comprehension of arguments by Chilean primary school students
Claudia Guerra and Romualdo Ibañez

BENNETT LECTURE THEATRE 2. Chair: Sarah J. White
Thematic session: Eye movement Control in Reading III

15:30 Reader targeting of words is guided by the distribution of information in the lexicon
Jon Carr and Davide Crepaldi

15:50 Prismatic glasses affect the binocular coordination during reading
Stephanie Jainta and Joëlle Joss

16:10 The Role of the Periphery in Comic Reading
Clare Kirtley, Christopher Murray, Phillip Vaughan and Benjamin Tatler

BENNETT LECTURE THEATRE 8. Chair: Zhanhan Tu
Thematic session: Pupillometry

15:30 What does the pupillary light response tell us about the mechanisms underlying object-based attention?
Yaffa Yeshurun, Felipe Luzardo and Wolfgang Einhäuser

15:50 Effects of luminance and arousal related baseline amplitude on the auditory phasic pupil dilation response
Andreas Widmann, Wolfgang Einhäuser, Nicole Wetzel and Phillip M. Alday

16:10 Warming up an eye tracker alleviates system drift in gaze position and pupil size
Richard Andersson and Kenneth Holmqvist

END OF PARALLEL SESSIONS

16.30 – 17.30 CLOSING PRESENTATION (BENNETT LECTURE THEATRE 1)

17.30 – 18.30 BENNETT LECTURE THEATRE 1

KEYNOTE: Monica S. Castelhano

Explorations of how scene context and previous experience dynamically influence attention and eye movement guidance

END OF CONFERENCE

Diagram of Bennett Building

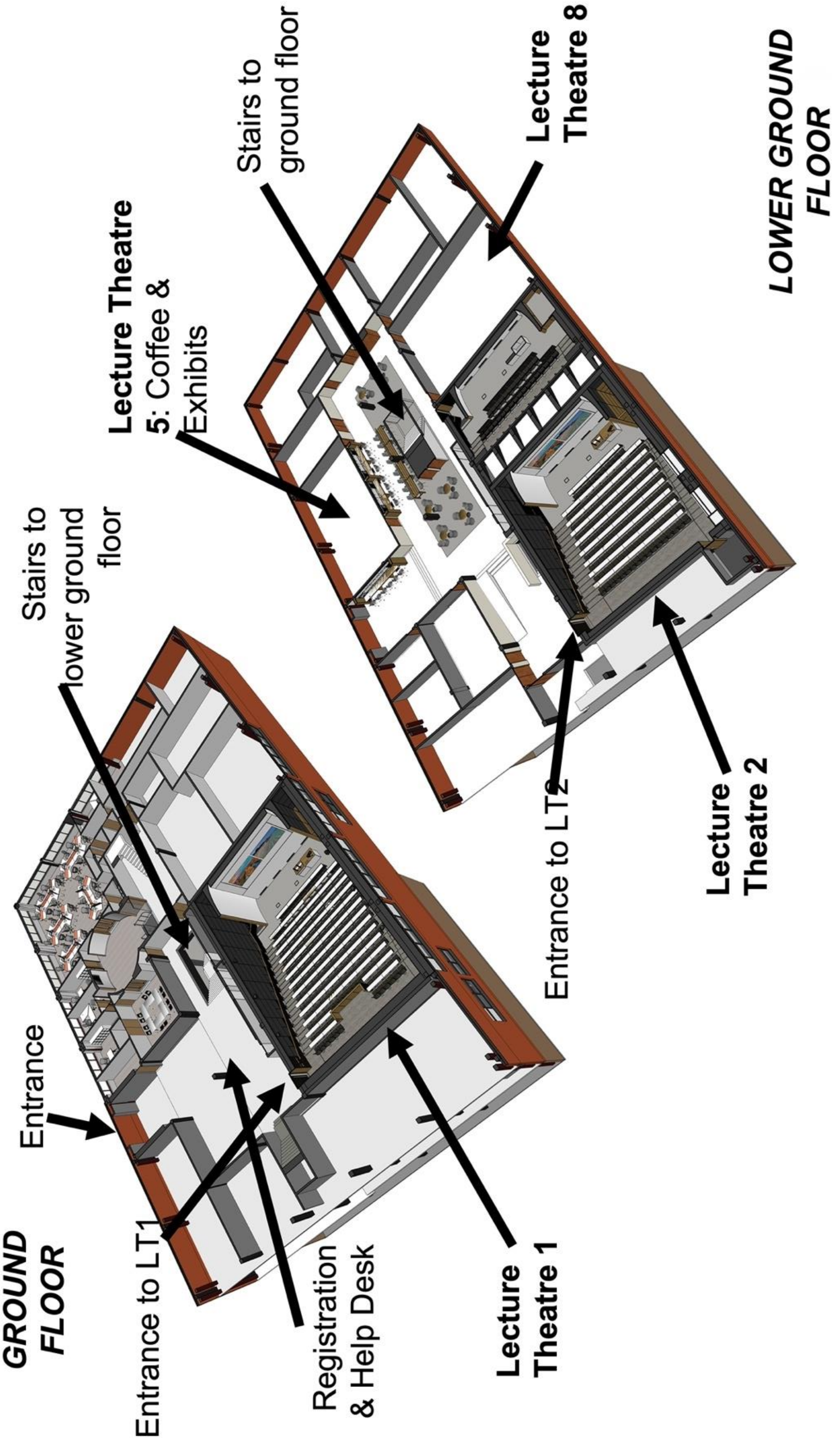

Author index

Abeles, Dekel, 40
Acarturk, Cengiz, 40
Acton, Jennifer, 34
Adedeji, Victoria, 25, 36
Adeli, Hossein, 35
Aispuriete, Inese, 49
Aizenshtein, Alina, 38
Albi, Ádám, 38
Alday, Phillip, 51
Alexander, Robert, 37
Alexeeva, Svetlana, 40
Aljassmi, Maryam, 33
Allen, Shanley, 40, 46
Almajed, Saad, 31
Alvarez, Marta, 31
Amiebenomo, Onyeka, 34
Andersson, Richard, 37, 51
Andrews, Sally, 28, 34
Angele, Bernhard, 42
Anisimov, Victor, 30
Antúnez, Martín, 27
Arco, Nicole, 28, 30
Areshenkoff, Corson, 28
Armstrong, Thomas, 37
Arnott, Stephen, 50
Arshad, Qadeer, 45
Artemenko, Elena, 29
Ashkenazi, Simone, 38
Atanasov, Petar, 49
Atkins, Andrew, 51
Babanova, Ksenia, 30
Bach, Michael, 34
Backhaus, Daniel, 38, 40
Bai, Xuejun, 25, 30, 31, 44, 45
Barach, Eliza, 49
Barber, Horacio, 27
Barrett, Doug, 7, 36
Barton, Hayley, 30
Bartseva, Ksenia, 30, 39
Basvitch, Itay, 28
Beaton, Derek, 29, 50
Bellerby, Emily, 39
Benedek, Mathias, 31
Benjamins, Jeroen, 25, 37, 39
Benson, Valerie, 20, 25, 29, 39, 46
Bényei, Gábor, 38
Berg, Esther, 29
Bertram, Raymond, 42
Bhanap, Ruhi, 38
Biliotti, Carolina, 31
Bills, Katherine, 30
Binns, Malcolm, 50
Black, Sandra, 29, 50
Blignaut, Pieter, 37
Blohm, Stefan, 42
Blythe, Hazel, 35, 36, 39
Bode, Lisa, 45
Böing, Sanne, 29, 38
Bompas, Aline, 35
Bonsu, Angela, 45
Booij, Linda, 50
Boudelaa, Sami, 33
Bramão, Inês, 44
Breen, David, 28
Breil, Christina, 41
Brenner, Eli, 40
Brien, Donald, 28, 29, 43, 50
Brink, Teuni, 29, 38
Broek, Gesa, 38
Brouwer, Anne, 37
Bruland, Rouwen, 45
Bruner, Emiliano, 25

Brunet, Milca, 39
Bucci, Maria, 27
Burgart, Ksenia, 40
Burle, Boris, 27
Byrne, Sean, 31
Cabañas, Cynthia, 49
Cabestrero, Raúl, 29
Calancie, Olivia, 43, 50
Caliskan, Neslihan, 39
Carr, Jon, 26, 51
Caruso, Letizia, 36
Caspi, Avi, 50
Casteau, Soazig, 45
Castelhano, Monica, 8, 20, 48, 52
Castet, Eric, 27
Catrysse, Leen, 28
Cepero, Laura, 29
Chamorro, Eva, 31
Chang, Min, 45
Chernova, Daria, 40
Chernova, Uliana, 29
Chernozatonskiy, Konstantin, 30
Chevet, Guillaume, 30
Chistofalos, Andriana, 49
Chiu, Tzu, 42
Christofalos, Andriana, 26, 30
Chuang, Lewis, 37
Clarke, Alasdair, 36
Cleva, José, 31
Coe, Brian, 28, 29, 43, 50
Conde, Susana, 37
Contreras, Mª, 29
Cooley, Frances, 46
Cordero, Ismael, 42
Cornelissen, Frans, 40, 50
Crawford, Trevor, 28, 30
Crepaldi, Davide, 26, 51
Cristino, Filipe, 50
Cui, Lei, 48
Dagit, Gerald, 27
Dalmaijer, Edwin, 31, 37
Dalrympleke, Kirsten, 37
Dare, Zoya, 27
Dayan, Oryah, 49
Degg, Christopher, 34
Degno, Federica, 27, 44, 49
Deković, Maja, 25
Dewar, Jane, 29
Dias, Elisa, 26
Divjak, Dagmar, 46
Doorn, Andrea, 25
Dotor, Paulina, 31
Dragoy, Olga, 30, 39, 40
Dreimane, Lana, 49
Dreneva, Anna, 29, 38, 40
Drieghe, Denis, 35, 37, 42, 48
Driessen, Joost, 40
Duke, Philip, 31
Dunn, Matt, 34, 37
Ede, Freek, 38, 44
Eekhof, Lynn, 42
Eggert, Thomas, 35
Einhäuser, Wolfgang, 51
Eldar, Eran, 49
Emmorey, Karen, 46
Engbert, Ralf, 38, 40
Ennis, Fergal, 34
Erdmann, Mirjamaija, 36, 39
Erichsen, Erichsen, 34
Erichsen, Jonathan, 34
Ermolova, Maria, 29
Essmann, Lucas, 43
Ettinger, Ulrich, 37
Faber, Myrthe, 42
Facevicova, Kamila, 38
Feldman, Laurie, 49
Fergusson, James, 34
Fernandez, Leigh, 40, 46
Ferrer, Xavier, 39
Fiedler, Susann, 37

Filik, Ruth, 49
Finger, Elizabeth, 29, 50
Fink, Lauren, 43
Fisher, Donald, 48
Fleming, Roland, 31
Fokin, Danil, 42
Fontoura, Pablo, 27
Foulsham, Tom, 37, 43
Freedman, Morris, 29, 50
Friede, Anne, 42
Fu, Ying, 31, 44
Furman, Reyhan, 30
Gago, Maria, 25
Garcia, Clara, 31
Gava, Giuseppe, 45
Geest, Jos, 37
Gesztesi, Gábor, 35
Ghasia, Fatema, 8, 18, 34
Ghiani, Andrea, 40
Gilchrist, Iain, 29
Gill, Donna, 25
Glandorf, Domink, 26
Gnetov, Daniil, 30
Godwin, Hayward, 35, 48
Goktepe, Nedim, 45
Goldina, Sofya, 30, 39, 40
Gonzalez, Amelia, 31
Gottlob, Irene, 34
Grande, Pablo, 31
Grappe, Amandine, 39
Green, Christine, 30
Greenberg, Shlomit, 40, 44
Grimes, David, 28
Guan, Zheng, 45
Guerra, Claudia, 51
Guillaume, Chevet, 49
Guo, Meihua, 39
Gutzeit, Julian, 31, 32
Haan, Gera, 40, 50
Hadwin, Julie, 25
Häikiö, Tuomo, 36, 39
Halbmayr, Helene, 29
Halonen, Sohvi, 36
Hansen, Dan, 37
Hao, Lisha, 45
Hautala, Jarkko, 34
He, Shiyu, 46
Hedge, Craig, 35
Heikkilä, Timo, 33, 48
Helmchen, Christoph, 45
Henderson, John, 20, 48
Hessels, Roy, 25, 37, 39
Heuer, Anna, 44
Heutink, Joost, 40, 50
Hodgson, Timothy, 25
Höfler, Margit, 29
Hofmann, Markus, 27
Holleman, Gijs, 25
Holmqvist, Kenneth, 37, 51
Hooge, Ignace, 25, 37, 39
Hoogerbrugge, Alex, 31, 38
Hoogerheide, Vincent, 38
Hout, Liz, 40
Howard, Philippa, 46
Hsieh, Ming, 40
Huang, Jeff, 28, 29, 50
Huestegge, Lynn, 31, 32, 38, 41
Hughes, Anna, 36
Huijding, Jorg, 25
Hunt, Amelia, 36
Hutton, Samuel, 37
Hyönä, Jukka, 33, 48
Ibañez, Romualdo, 51
Indurkhya, Bipin, 40
Inhoff, Albrecht, 42
Ioannidou, Flora, 25
Irvine, Alexander, 37
Irwin, Amy, 36
Isel, Frédéric, 27
Iskra, Ekaterina, 40

Itti, Laurent, 43
Ivanov, Michael, 40, 28
Jainta, Stephanie, 32, 51
Jarodzka, Halszka, 28
Javitt, Daniel, 26
Jensen, Kåre, 39
Jia, Man, 38
Jian, Yu, 39
Jiskoot, Lize, 29
Jog, Mandar, 28
Johansson, Mikael, 44
Johansson, Roger, 44
Jones, Beth, 50
Joss, Joëlle, 32, 51
Judge, Jeannie, 25
Kaakinen, Johanna, 39, 42
Kalabusova, Veronika, 38
Kalkan, Sinan, 40
Kalsi, Tami, 49
Kaltenbach, Katharina, 35
Kanerva, Oksana, 36, 39
Kaprielova, Anastasiia, 30, 39
Kasneci, Enkelejda, 37
Kekunnaya, Ramesh, 50
Kemner, Chantal, 25, 39
Kerckhoffs, Andrienne, 28
Khan, Sarosh, 50
Kim, Junhui, 31
Kingstone, Alan, 37
Kirkby, Julie, 25, 30, 36
Kirkden, Richard, 27
Kirkpatrick, Ryan, 43
Kirtley, Clare, 51
Knox, Paul, 37
Koenderink, Jan, 25
Koerfer, Krischan, 45
Kok, Ellen, 38
Koltsova, Olessia, 29
König, Peter, 40, 43
König, Sabine, 43
Konovalova, Anastasiia, 30
Korda, Ziva, 31
Korepanov, Andrew, 39
Korhummel, Carola, 27
Körner, Christof, 29, 31
Kosikova, Alisa, 28
Kreyenmeier, Philipp, 44, 45
Krieken, Kobie, 42
Kroell, Lisa, 44
Krutz, Alexander, 28
Kucharský, Šimon, 37
Kuht, Helen, 34
Kuo, Pin, 39
Kuperman, Victor, 30, 35
Kürten, Jens, 31, 32
Kwan, Donna, 28, 29, 50
Ladinskaya, Nina, 30, 39
Laks, Madison, 26, 30
Lan, Zebo, 39
Landmann, Eva, 41
Lang, Anthony, 28, 29, 50
Lange, Elke, 43
Langlois, Annick, 44
Lappe, Markus, 35, 45
Latanov, Alexander, 38
Latyshkova, Alexandra, 30
Laubrock, Jochen, 28, 36
Ledgeway, Timothy, 31
Lee, Charlotte, 35, 48
Lee, Helena, 37
Lee, Joy, 37
Leppänen, Jukka, 37
Levine, Brian, 28, 50
Li, Jie, 38
Li, Xingshan, 48
Li, Xiyuan, 45
Lin, Juntian, 38
Lin, Sunny, 40, 45
Liu, Baiwei, 38
Liu, Nina, 39, 45

Liu, Zhong, 44
Liversedge, Simon, 14, 18, 20, 25, 27, 30, 31, 36, 39, 44, 45, 46, 48, 49
Lizunkova, Kseniya, 38
Lopukhina, Anastasiya, 30, 39, 40
Lou, Wendy, 50
Lowman, Michael, 30
Lu, Zijia, 30
Lüken, Malte, 37
Luzardo, Felipe, 51
Lyapunov, Ivan, 28
Lyapunov, Sergey, 28
Machner, Bjoern, 45
MacInnes, Joseph, 29
Macknik, Stephen, 37
Majaranta, Päivi, 37
Mak, Marloes, 42
Malsburg, Titus, 26
Mancini, Régis, 27
Manning, Hannah, 36
Manoli, Athina, 25
Manzanera, Antoine, 35
Marcet, Ana, 42, 48
Marras, Connie, 28, 29, 50
Masellis, Mario, 28, 50
Maturi, Kinnera, 28
Mayas, Julia, 29
McArthur, Genevieve, 26
McCarty, Kristofor, 39
McCloy, Rachel, 35
McGowan, Victoria, 2, 30
McGraw, Paul, 31
McIlreavy, Lee, 34
McLaughlin, Paula, 28, 29, 50
McLean, Rebecca, 34
McSorley, Eugene, 35
Mecklenbeck, Michael, 37
Meghanathan, Radha, 25
Meisinger, Elizabeth, 36
Meixner, Johannes, 36
Méndez, Laura, 29
Meng, Michael, 29
Mézière, Diane, 26
Milin, Petar, 46
Millan, Jorge, 37
Milledge, Sara, 36, 39
Milligan, Sara, 27, 39
Minh, Alexandra, 39
Mirault, Jonathan, 46
Mironets, Sofia, 40
Miscena, Anna, 27
Momose, Keiko, 32
Montoro, Pedro, 29
Moosa, Maleeha, 44
Morozov, Anna, 40
Müller, Katharina, 25
Munoz, Douglas, 28, 29, 43, 50
Murray, Christopher, 51
Nahari, Tal, 49
Nawrocki, Piotr, 40
Nejad, Ashkan, 40, 50
Niedra, Laima, 49
Niehorster, Diederick, 37, 39
Nijboer, Tanja, 31, 38
Nikolaev, Andrey, 44
Nilsson, Mattias, 36
Nolte, Debora, 40
Norkina, Marina, 40
Nowakowska, Anna, 36
Nuthmann, Antje, 37
Nyström, Marcus, 37, 39
Oberauer, Klaus, 38
Ohl, Sven, 44
Olah, Zoril, 31
Olkoniemi, Henri, 36
Orange, J.B., 28, 50
Örbom, Saga, 37
Orenes, Isabel, 29
Orquin, Jacob, 37
Ossandón, José, 50

Pagán, Ascensión, 2, 27, 45, 46, 51
Pajkossy, Péter, 35, 38
Palol, Marc, 40
Pamplona, Daniela, 35
Papenmeier, Frank, 27
Paranko, Birgitta, 48
Park, Soon, 37
Parker, Jessica, 43
Parmentier, Fabrice, 30
Parshina, Olga, 40
Paterson, Kevin, 2, 7, 27, 30, 33, 35, 45, 49
Pavlou, Katerina, 25
Pedersen, Nadia, 39
Pedrero, José, 31
Peltsch, Alicia, 28
Perea, Manuel, 33, 42, 48
Perkins, Julia, 43
Perra, Joris, 31
Pertzov, Yoni, 49
Pescuma, Valentina, 26
Petrova, Tatiana, 40
Pexman, Penny, 36
Pieczykolan, Aleks, 38
Pikunov, Andrey, 30
Plunkett, Anna, 30
Polden, Megan, 30
Pollmann, Stefan, 25
Polonio, Luca, 31
Poos, Jackie, 29
Popelka, Stanislav, 37, 38, 43
Postuma, Eva, 40, 50
Potthoff, Jonas, 29
Premeti, Aikaterini, 27
Prichard, Caleb, 51
Prieto, Antonio, 29
Proudlock, Frank, 7, 34, 37
Qahtani, Fawziah, 49
Raab, Markus, 45
Radach, Ralph, 5, 27, 33, 42, 49
Raettig, Anne, 41
Raettig, Tim, 32
Raijmakers, Maartje, 29
Rancz, Ede, 39
Ranta, Emilia, 42
Reichle, Erik, 26, 34, 35
Reitstätter, Luise, 27
Renkewitz, Frank, 37
Reuther, Josephine, 36
Riccaboni, Massimo, 31
Richards, Simon, 46
Riek, Heidi, 28, 43, 50
Roberts, Angela, 28, 50
Rodán, Antonio, 29
Röder, Brigitte, 50
Rodrigues, Erica, 42
Rolfs, Martin, 44
Rölke, Andre, 27
Roorda, Austin, 37
Rosenbaum, David, 36
Rosenberg, Raphael, 27
Rosner, Agnes, 38
Ryan, Jennifer, 44
Sajjid, Asha, 30
Sanders, José, 42
Sauerberger, Kyle, 36
Saunders, Emily, 46
Saxena, Shreshth, 43
Scheepers, Christoph, 46
Schienle, Anne, 29
Schluppeck, Denis, 31
Schönfelder, Petra, 29
Schotter, Elizabeth, 27, 34, 39, 46
Schroeder, Sascha, 26, 30, 31, 42, 49
Schroeger, Anna, 45
Schütz, Alexander, 31, 45
Schwalm, Laura, 33, 49
Schwan, Stephan, 27
Schwetlick, Lisa, 38

Seelaar, Harro, 29
Seernani, Divya, 39
Senju, Atsushi, 49
Serebryakova, Margarita, 40
Shareef, Idris, 50
Sharif, Bonita, 37
Sharif, Mishaal, 45
Shen, Kelly, 44
Sheridan, Heather, 26, 28, 30, 49
Sherman, Charli, 36
Sheth, Viral, 34
Shic, Frederick, 37
Shoshina, Irina, 28
Shovman, Mark, 37, 40
Shurupova, Marina, 38, 40
Simola, Jaana, 42
Simon, Yuliya, 28
Slattery, Timothy, 25, 36
Smith, Daniel, 45
Smith, Tim, 49
Smorenburg, Matthew, 29, 43
Sniezynski, Bartlomiej, 40
Sobolewska, Aleksandra, 29
Souto, David, 2, 7, 31
Spering, Miriam, 19, 23, 35, 44, 45
Spethmann, Sebastian, 28
Spieser, Laure, 27
Sprenger, Andreas, 45
Stamkou, Eftychia, 29
Stanovaya, Viktoriya, 28
Staroverova, Vladislava, 30, 39, 40
Startsev, Mikhail, 37
Steeves, Thomas, 28
Stewart, Emma, 31
Stigchel, Stefan, 29, 31, 38
Stoffi, Falco, 31
Straube, Andreas, 35
Strauch, Christoph, 31
Strobl, Josef, 43
Strong, Michael, 29
Strother, Stephen, 29, 50
Sturt, Patrick, 42
Sui, Longjiao, 39
Sultan, Minha, 43
Sumner, Petroc, 35
Sunderland, Kelly, 50
Swartz, Richard, 28, 29, 50
Takemoto, Ayumi, 49
Tan, Brian, 28, 29, 50
Tartaglia, Carmela, 29, 50
Tas, Caglar, 43
Tatiana, Petrova, 30
Tatler, Benjamin, 51
Taub, Keren, 44
Teixeira, Elisangela, 42
Telgmann, Leonie, 25
Terpilovskii, Maksim, 29
Thomas, Mervyn, 7, 34, 37
Thomas, Nikita, 34
Tilmatine, Mesian, 42
Timmis, Matthew, 28
Tompson, Meyrem, 30
Troyer, Angela, 28, 50
Tu, Zhanhan, 34, 50, 51
Tze, Natalie, 42
Ulianova, Taisiia, 29
Vakil, Eli, 38
Valtakari, Niilo, 39
Vasilev, Martin, 25, 30, 36
Vaughan, Phillip, 51
Vedenina, Olga, 30, 39
Veldre, Aaron, 28, 34
Venrooij, Ward, 37
Vieira, Joao, 42
Visser, Ingmar, 37
Vitu, Françoise, 27, 35
Vorstius, Christian, 27, 42, 49
Vziatysheva, Victoria, 29
Wagner, Christoph, 27
Wagner, Ilja, 31

Walcher, Sonja, 31
Walker, Robin, 45
Walter, Jasmin, 43
Wang, Jingxin, 36, 45
Wang, Mengsi, 25, 44
Wang, Shuangshuang, 45
Wang, Yongsheng, 45
Ward, Katherine, 34
Warrington, Kayleigh, 35, 45, 49
Waters, Lee, 28
Watson, Jonathan, 30
Watson, Tamara, 45
Wei, Lixin, 44
Weller, Lisa, 31
Wetzel, Nicole, 51
White, Brian, 43
White, Sarah, 27, 30, 35, 49, 51
Widmann, Andreas, 51
Willems, Roel, 42
Williams, Kirsten, 31
Wilson, Jessica, 39
Wolf, Christian, 35
Wolf, Kerstin, 39
Wolfer, Stephanie, 26
Wong, Roslyn, 28
Woodhouse, J, 34
Wu, Sarah, 30
Wu, Shihui, 35
Wynn, Jordana, 44
Xheladini, Agnesa, 40
Xie, Hongyu, 36
Xie, Mingqing, 42
Xu, Yuening, 29
Yan, Guoli, 29, 30, 31, 39, 45
Yang, Fuyi, 29
Yep, Rachel, 28, 43
Yeshurun, Yaffa, 51
Yoshida, Takako, 31
Yoshinari, Yuko, 40
Yu, Lili, 26, 34
Yuan, Xiaoyuan, 39
Zang, Chuanli, 25, 30, 31, 39, 44, 45
Zdorova, Nina, 30, 39, 40
Zehngut, Amit, 40
Zelinsky, Gregory, 35
Zemblys, Raimondas, 37
Zentall, Thomas, 36
Zerbib, Véronique, 30, 31, 49
Zerr, Paul, 50
Zhang, Kuo, 45
Zhang, Li, 29
Zhang, Manman, 44
Zhang, Qi, 38
Zhang, Yu, 38
Zhang, Zhichao, 30
Zhigulskaya, Daria, 30
Zhou, Li, 29
Zinman, Lorne, 29, 50
Zonneveld, Anne, 38

Exhibitors and Sponsors

Gold Sponsors

SR Research EyeLink®

tobii pro

Silver Sponsors

Pupil Labs

Inseye

IMOTIONS®
Unpack Human Behavior

Bronze Sponsors

EYESEETEC

5

1

0

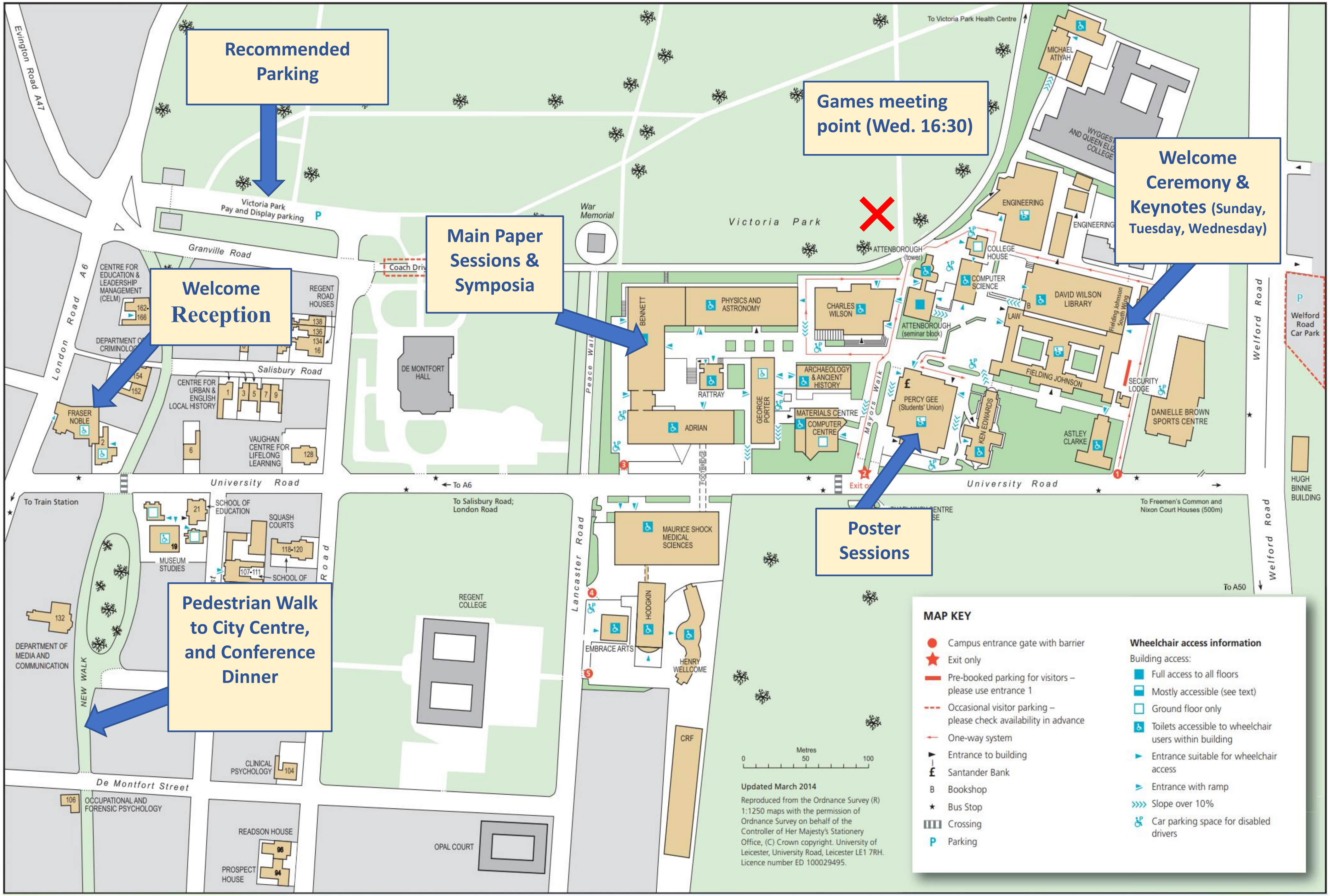

Recommended Parking
Welcome Reception
Main Paper Sessions & Symposia
Games meeting point (Wed. 16:30)
Welcome Ceremony & Keynotes (Sunday, Tuesday, Wednesday)
Poster Sessions
Pedestrian Walk to City Centre, and Conference Dinner
Evington Road A47
London Road A6
Granville Road
Victoria Park Pay and Display parking
Salisbury Road
University Road
Lancaster Road
De Montfort Street
Welford Road
Victoria Park
War Memorial
Peace Walk
Mayors Walk
New Walk
To Train Station
To Victoria Park Health Centre
To A6
To Salisbury Road; London Road
To Freemen's Common and Nixon Court Houses (500m)
To A50
Welford Road Car Park
Coach Driv
CENTRE FOR EDUCATION & LEADERSHIP MANAGEMENT (CELM)
DEPARTMENT OF CRIMINOLO
FRASER NOBLE
REGENT ROAD HOUSES
CENTRE FOR URBAN & ENGLISH LOCAL HISTORY
VAUGHAN CENTRE FOR LIFELONG LEARNING
DE MONTFORT HALL
BENNETT
PHYSICS AND ASTRONOMY
CHARLES WILSON
ATTENBOROUGH (tower)
ATTENBOROUGH (seminar block)
COLLEGE HOUSE
COMPUTER SCIENCE
ENGINEERING
MICHAEL ATIYAH
WYGGESTON AND QUEEN ELIZ COLLEGE
DAVID WILSON LIBRARY
Fielding Johnson South Wing
LAW
FIELDING JOHNSON
SECURITY LODGE
DANIELLE BROWN SPORTS CENTRE
ASTLEY CLARKE
KEN EDWARDS
PERCY GEE (Students' Union)
ARCHAEOLOGY & ANCIENT HISTORY
MATERIALS CENTRE
COMPUTER CENTRE
GEORGE PORTER
RATTRAY
ADRIAN
FOOTBRIDGE
MAURICE SHOCK MEDICAL SCIENCES
HODGKIN
EMBRACE ARTS
HENRY WELLCOME
CRF
REGENT COLLEGE
OPAL COURT
HUGH BINNIE BUILDING
SCHOOL OF EDUCATION
SQUASH COURTS
MUSEUM STUDIES
SCHOOL OF
DEPARTMENT OF MEDIA AND COMMUNICATION
CLINICAL PSYCHOLOGY
OCCUPATIONAL AND FORENSIC PSYCHOLOGY
READSON HOUSE
PROSPECT HOUSE
Exit o
Metres 0 50 100
Updated March 2014
Reproduced from the Ordnance Survey (R) 1:1250 maps with the permission of Ordnance Survey on behalf of the Controller of Her Majesty's Stationery Office, (C) Crown copyright. University of Leicester, University Road, Leicester LE1 7RH. Licence number ED 100029495.
MAP KEY
Campus entrance gate with barrier
Exit only
Pre-booked parking for visitors – please use entrance 1
Occasional visitor parking – please check availability in advance
One-way system
Entrance to building
Santander Bank
Bookshop
Bus Stop
Crossing
Parking
Wheelchair access information
Building access:
Full access to all floors
Mostly accessible (see text)
Ground floor only
Toilets accessible to wheelchair users within building
Entrance suitable for wheelchair access
Entrance with ramp
Slope over 10%
Car parking space for disabled drivers
